# Dataset of breath research manuscripts curated using PubMed search strings from 1995–2016

**DOI:** 10.1016/j.dib.2018.04.063

**Published:** 2018-05-02

**Authors:** M. Ariel Geer Wallace, Joachim D. Pleil

**Affiliations:** US Environmental Protection Agency, Office of Research and Development, National Exposure Research Laboratory, 109 T.W. Alexander Drive, Research Triangle Park, NC 27711, USA

**Keywords:** Exhaled breath, Exhaled breath condensate (EBC), Exhaled breath aerosol (EBA), Mass spectrometry, Sensors, Immunochemistry, Public health, Clinical diagnosis

## Abstract

The data contained in this article are PubMed search strings and search string builders used to curate breath research manuscripts published from 1995–2016 and the respective number of articles found that satisfied the search requirements for selected categories. Breath sampling represents a non-invasive technique that has gained usefulness for public health, clinical, diagnostic, and environmental exposure assessment applications over the years. This data article includes search strings that were utilized to retrieve publications through the PubMed database for different breath research-related topics that were related to the analysis of exhaled breath, exhaled breath condensate (EBC), and exhaled breath aerosol (EBA) as well as the analysis of cellular headspace. Manuscripts were curated for topics including EBC, EBA, Direct MS, GC–MS, LC-MS, alcohol, and sensors. A summary of the number of papers published per year for the data retrieved using each of the search strings is also included. These data can be utilized to discern trends in the number of breath research publications in each of the different topics over time. A supplementary Appendix A containing the titles, author lists, journal names, publication dates, PMID numbers, and EntrezUID numbers for each of the journal articles curated using the finalized search strings for the seven breath research-related topics can also be found within this article. The selected manuscripts can be used to explore the impact that breath research has had on expanding the scientific knowledge in each of the investigated topics.

**Specifications Table**

**Table:** 

Subject area	*Breath research, database searching, public health*
More specific subject area	*Gas-phase exhaled breath, exhaled breath condensate, exhaled breath aerosol, volatile organic compound (VOC) analysis, instrumentation, pulmonary disorders, community assessment*
Type of data	*Tables and Spreadsheets*
How data was acquired	*PubMed database searches*
Data format	*Filtered*
Experimental factors	*The PubMed search strings were built for seven main exhaled breath research topics: exhaled breath aerosol, exhaled breath condensate, Direct MS, GC–MS, LC-MS, alcohol, and sensors. The searches for all topics except alcohol were restricted from 1995/01/01 until 2016/12/31; alcohol was restricted from 01/01/1960 to 12/31/2016. Restrictions were included to eliminate topics related to medical diagnosis that did not include evaluation of exhaled breath.*
Experimental features	*The PubMed search strings were utilized to acquire journal articles related to different breath research topics that include the analysis of exhaled breath, EBC, EBA, or cellular headspace. The number of publications per year for each topic is included as well as an appendix listing the journal article curated for each topic area.*
Data source location	*The data were gathered from PubMed using the PubMed Advanced Search Builder:*https://www.ncbi.nlm.nih.gov/pubmed/advanced.
Data accessibility	*The data is contained within this article and the attached Appendix.*
Related research article	*Wallace, M. A. G. and Pleil, J. D., Evolution of Clinical and Environmental Health Applications of Exhaled Breath Research: Review of Methods and Instrumentation for Gas-phase, Condensate, and Aerosols. Analytica Chimica Acta **2018**, Submitted.*

**Value of the data**

• Researchers can use the acquired lists of breath research publications to gather references for review of the literature.
• Researchers can use the data to evaluate trends in the number and type of breath research manuscripts published from 1995–2016.
• Researches will be able to understand the value of exhaled breath research and the impact it has had on improving public health and the community through medical (clinical, diagnostic) and environmental (exposure, adverse outcome) applications.
• Researchers can use the developed search strings to curate additional breath research publications within the time frame, expand the time frame, or remove restrictions to obtain different lists of publications for assessment and review.

## 1 Data

The presented data are six tables and a supplementary Appendix file. Table 1 contains the initial PubMed search string builders that were developed for the individual breath-related topics that were used to curate papers. These search string builders include “exhaled breath,” “VOCs and headspace,” “biological media,” “aerosols,” “condensate,” “Direct MS,” “GC–MS,” “LC-MS,” “alcohol,” and “sensors.” Table 2 contains combinations of the individual search strings listed in Table 1. These search strings were developed to ensure that the retrieved publications were focused on the subject of exhaled breath research and volatile compound analysis. Restrictions were introduced to the “exhaled breath” search strings in order to eliminate papers that were found to be out of scope after reviewing the search results obtained using the search string in Table 1. Many of the papers that were out of scope in the initial search were from medical journals describing the health state of patients, but exhaled breath, condensate, and aerosol were not evaluated in these studies. Table 3 presents the final search strings for the topic areas evaluated, which include combinations of the search string builders included in Table 1, Table 2. Publications were curated for the breath research-related topics listed in Table 3: EBA, EBC, Direct MS, GC–MS, LC-MS, alcohol, and sensors. For Direct MS, GC–MS, LC-MS, and sensors, the search strings of “VOCs and headspace” and “biological media” were included to retrieve articles related to cellular headspace and the analysis of biological fluids using mass spectrometry and sensor instrumentation.

**Table 1:** Level 1 PubMed Search String Builders developed using the PubMed Advanced Search Builder. Ten search string builders for topics related to exhaled breath research are listed. The searches were date restricted from 1995–2016 for all topics except alcohol, which was searched from 1960–2016. The number of publications retrieved using each of these search strings is shown in the table.

Search Number	Topic Area	PubMed Advanced Search Builder	Number of Publications
1	Exhaled Breath	((breath[Title/Abstract]) OR exhaled air[Title/Abstract]) AND ("1995/01/01"[Date - Publication]: "2016/12/31"[Date - Publication])	28,183
2	VOCs and Headspace	(((volatile organic compound[Title/Abstract]) OR VOC[Title/Abstract]) OR headspace[Title/Abstract]) AND ("1995/01/01"[Date - Publication]: "2016/12/31"[Date - Publication])	9404
3	Biological Media	(((((((((blood[Title/Abstract]) OR urine[Title/Abstract]) OR feces[Title/Abstract]) OR faeces[Title/Abstract]) OR milk[Title/Abstract]) OR cell line[Title/Abstract]) OR cell culture[Title/Abstract]) OR bacteria[Title/Abstract]) OR saliva[Title/Abstract]) AND ("1995/01/01"[Date - Publication]: "2016/12/31"[Date - Publication])	1,571,597
4	Aerosols	(((((((aerosol[Title/Abstract]) OR aerosols[Title/Abstract]) OR particle[Title/Abstract]) OR particles[Title/Abstract]) OR exhaled breath aerosol[Title/Abstract]) OR EBA[Title/Abstract])) AND ("1995/01/01"[Date - Publication]: "2016/12/31"[Date - Publication])	215,189
5	Condensate	(((condensate[Title/Abstract]) OR exhaled breath condensate[Title/Abstract]) OR EBC[Title/Abstract]) AND ("1995/01/01"[Date - Publication]: "2016/12/31"[Date - Publication])	4457
6	Direct MS	((((((((((proton transfer reaction mass spectrometry[Title/Abstract]) OR PTR-MS[Title/Abstract]) OR proton transfer reaction time-of-flight mass spectrometry[Title/Abstract]) OR PTR-TOF[Title/Abstract]) OR SIFT-MS[Title/Abstract]) OR selected ion flow tube mass spectrometry[Title/Abstract]) OR ion mobility spectrometry[Title/Abstract]) OR ion mobility mass spectrometry[Title/Abstract]) OR IMS-MS[Title/Abstract]) OR IMS[Title/Abstract]) AND ("1995/01/01"[Date - Publication]: "2016/12/31"[Date - Publication])	4,984
7	GC–MS	(((((((((((((((((((gas chromatography-mass spectrometry[Title/Abstract]) OR GC–MS[Title/Abstract]) OR GC/MS[Title/Abstract]) OR GC–MS/MS[Title/Abstract]) OR GCxGC[Title/Abstract]) OR GCxGC-MS[Title/Abstract]) OR gas chromatography time-of-flight mass spectrometry[Title/Abstract]) OR GC-TOF-MS[Title/Abstract]) OR GC-TOF[Title/Abstract]) OR (gas chromatography[Title/Abstract] AND mass spectrometry[Title/Abstract])) OR (automated thermal desorption[Title/Abstract] AND gas chromatography[Title/Abstract])) OR ATD-GC/MS[Title/Abstract]) OR (ATD[Title/Abstract] AND GC–MS[Title/Abstract])) OR (orbitrap[Title/Abstract] AND GC[Title/Abstract])) OR (orbitrap[Title/Abstract] AND gas chromatography[Title/Abstract])) OR (QExactive[Title/Abstract] AND GC[Title/Abstract])) OR (QExactive[Title/Abstract] AND gas chromatography[Title/Abstract])) OR (ion trap[Title/Abstract] AND GC[Title/Abstract])) OR (ion trap[Title/Abstract] AND gas chromatography[Title/Abstract])) AND ("1995/01/01"[Date - Publication]: "2016/12/31"[Date - Publication])	40,219
8	LC-MS	(((((((((((((((((((((((((((((liquid chromatography-mass spectrometry[Title/Abstract]) OR LC-MS[Title/Abstract]) OR LC/MS[Title/Abstract]) OR LC-MS/MS[Title/Abstract]) OR (liquid chromatography[Title/Abstract] AND mass spectrometry[Title/Abstract])) OR liquid chromatography time-of-flight mass spectrometry[Title/Abstract]) OR LC-TOF-MS[Title/Abstract]) OR ESI-MS[Title/Abstract]) OR electrospray ionization mass spectrometry[Title/Abstract]) OR high performance liquid chromatography-mass spectrometry[Title/Abstract]) OR HPLC-MS[Title/Abstract]) OR ultrahigh performance liquid chromatography-mass spectrometry[Title/Abstract]) OR UHPLC-MS[Title/Abstract]) OR ultrahigh performance liquid chromatography-high resolution mass spectrometry[Title/Abstract]) OR UHPLC-HRMS[Title/Abstract]) OR (UHPLC[Title/Abstract] AND MS[Title/Abstract])) OR (HPLC[Title/Abstract] AND MS[Title/Abstract])) OR (nanoelectrospray[Title/Abstract] AND mass spectrometry[Title/Abstract])) OR (ion trap[Title/Abstract] AND LC[Title/Abstract])) OR (ion trap[Title/Abstract] AND liquid chromatography[Title/Abstract])) OR (ion trap[Title/Abstract] AND LC[Title/Abstract])) OR (quadrupole[Title/Abstract] AND LC[Title/Abstract])) OR (quadrupole[Title/Abstract] AND liquid chromatography[Title/Abstract])) OR Q-TOF[Title/Abstract]) OR quadrupole-time-of-flight[Title/Abstract]) OR (orbitrap[Title/Abstract] AND LC[Title/Abstract])) OR (orbitrap[Title/Abstract] AND liquid chromatography[Title/Abstract])) OR (QExactive[Title/Abstract] AND LC[Title/Abstract])) OR (QExactive[Title/Abstract] AND liquid chromatography[Title/Abstract])) AND ("1995/01/01"[Date - Publication]: "2016/12/31"[Date - Publication])	88,764
9	Sensors	(((((((((((((((electronic nose[Title/Abstract]) OR enose[Title/Abstract]) OR e-nose[Title/Abstract]) OR quantum cascade laser[Title/Abstract]) OR QCL[Title/Abstract]) OR fourier transform infrared spectroscopy[Title/Abstract]) OR FTIR[Title/Abstract]) OR non dispersive infrared sensor[Title/Abstract]) OR NDIR[Title/Abstract]) OR chemical array[Title/Abstract]) OR mid-infrared sensor[Title/Abstract]) OR sensor[Title/Abstract]) OR laser spectroscopy[Title/Abstract]) OR diode[Title/Abstract]) OR photoacoustic[Title/Abstract]) AND ("1995/01/01"[Date - Publication]: "2016/12/31"[Date - Publication])	115,762
10	Alcohol	((alcohol[Title/Abstract]) AND ethanol[Title/Abstract]) AND ("1960/01/01"[Date - Publication]: "2016/12/31"[Date - Publication])	22,045

**Table 2:** Level 2 Combined Search String Builders. This table contains combinations of the search string builders listed in Table 1 and several search strings contain restrictions, which are “NOT” statements that excluded papers unrelated to the analysis of exhaled breath. These search string builders were used as templates to create the final search strings shown in Table 3.

Search Number	Topic Area	PubMed Advanced Search Builder	Number of Publications
11	VOCs and Headspace (2) + Biological Media (3)	(((((volatile organic compound[Title/Abstract]) OR VOC[Title/Abstract]) OR headspace[Title/Abstract]) AND ("1995/01/01"[Date - Publication]: "2016/12/31"[Date - Publication]))) AND ((((((((((blood[Title/Abstract]) OR urine[Title/Abstract]) OR feces[Title/Abstract]) OR faeces[Title/Abstract]) OR milk[Title/Abstract]) OR cell line[Title/Abstract]) OR cell culture[Title/Abstract]) OR bacteria[Title/Abstract]) OR saliva[Title/Abstract]) AND ("1995/01/01"[Date - Publication]: "2016/12/31"[Date - Publication]))	1417
12	Exhaled Breath (1) + Restrictions	(((((((breath[Title/Abstract]) OR exhaled air[Title/Abstract]) AND ("1995/01/01"[Date - Publication]: "2016/12/31"[Date - Publication]))) NOT short of breath[Title/Abstract]) NOT shortness of breath[Title/Abstract]) NOT breath sounds[Title/Abstract]) NOT gastric emptying[Title/Abstract]	22,079
13	Exhaled Breath (1) + VOCs and Headspace (2) + Biological Media (3)	(((((((volatile organic compound[Title/Abstract]) OR VOC[Title/Abstract]) OR headspace[Title/Abstract]) AND ("1995/01/01"[Date - Publication]: "2016/12/31"[Date - Publication]))) AND ((((((((((blood[Title/Abstract]) OR urine[Title/Abstract]) OR feces[Title/Abstract]) OR faeces[Title/Abstract]) OR milk[Title/Abstract]) OR cell line[Title/Abstract]) OR cell culture[Title/Abstract]) OR bacteria[Title/Abstract]) OR saliva[Title/Abstract]) AND ("1995/01/01"[Date - Publication]: "2016/12/31"[Date - Publication])))) OR (((breath[Title/Abstract]) OR exhaled air[Title/Abstract]) AND ("1995/01/01"[Date - Publication]: "2016/12/31"[Date - Publication]))	29,775
14	Exhaled Breath (1) + VOCs and Headspace (2) + Biological Media (3) + Restrictions	((((((((((((volatile organic compound[Title/Abstract]) OR VOC[Title/Abstract]) OR headspace[Title/Abstract]) AND ("1995/01/01"[Date - Publication]: "2016/12/31"[Date - Publication]))) AND ((((((((((blood[Title/Abstract]) OR urine[Title/Abstract]) OR feces[Title/Abstract]) OR faeces[Title/Abstract]) OR milk[Title/Abstract]) OR cell line[Title/Abstract]) OR cell culture[Title/Abstract]) OR bacteria[Title/Abstract]) OR saliva[Title/Abstract]) AND ("1995/01/01"[Date - Publication]: "2016/12/31"[Date - Publication])))) OR (((breath[Title/Abstract]) OR exhaled air[Title/Abstract]) AND ("1995/01/01"[Date - Publication]: "2016/12/31"[Date - Publication])))) NOT short of breath[Title/Abstract]) NOT shortness of breath[Title/Abstract]) NOT breath sounds[Title/Abstract]) NOT gastric emptying[Title/Abstract]	23,377

**Table 3:** Level 3 Final Search Strings for All Topics Searched Using PubMed. This table contains combinations of the search strings shown in Table 1, Table 2 that were utilized to curate papers related to specific topics of interest to exhaled breath research or VOCs and biological headspace.

Search Number	Topic Area	Combined Search Builders	PubMed Advanced Search String	Number of Publications
15	EBA	Exhaled Breath + Restrictions + Aerosols	(((((((((breath[Title/Abstract]) OR exhaled air[Title/Abstract]) AND ("1995/01/01"[Date - Publication]: "2016/12/31"[Date - Publication]))) NOT short of breath[Title/Abstract]) NOT shortness of breath[Title/Abstract]) NOT breath sounds[Title/Abstract]) NOT gastric emptying[Title/Abstract])) AND ((((((((aerosol[Title/Abstract]) OR aerosols[Title/Abstract]) OR particle[Title/Abstract]) OR particles[Title/Abstract]) OR exhaled breath aerosol[Title/Abstract]) OR EBA[Title/Abstract])) AND ("1995/01/01"[Date - Publication]: "2016/12/31"[Date - Publication]))	535
16	EBC	Exhaled Breath + Restrictions + Condensate	(((((((((breath[Title/Abstract]) OR exhaled air[Title/Abstract]) AND ("1995/01/01"[Date - Publication]: "2016/12/31"[Date - Publication]))) NOT short of breath[Title/Abstract]) NOT shortness of breath[Title/Abstract]) NOT breath sounds[Title/Abstract]) NOT gastric emptying[Title/Abstract])) AND ((((condensate[Title/Abstract]) OR exhaled breath condensate[Title/Abstract]) OR EBC[Title/Abstract]) AND ("1995/01/01"[Date - Publication]: "2016/12/31"[Date - Publication]))	1327
17	Direct MS	Exhaled Breath + VOCs and Headspace + Biological Media + Restrictions + Direct MS	((((((((((((((volatile organic compound[Title/Abstract]) OR VOC[Title/Abstract]) OR headspace[Title/Abstract]) AND ("1995/01/01"[Date - Publication]: "2016/12/31"[Date - Publication]))) AND ((((((((((blood[Title/Abstract]) OR urine[Title/Abstract]) OR feces[Title/Abstract]) OR faeces[Title/Abstract]) OR milk[Title/Abstract]) OR cell line[Title/Abstract]) OR cell culture[Title/Abstract]) OR bacteria[Title/Abstract]) OR saliva[Title/Abstract]) AND ("1995/01/01"[Date - Publication]: "2016/12/31"[Date - Publication])))) OR (((breath[Title/Abstract]) OR exhaled air[Title/Abstract]) AND ("1995/01/01"[Date - Publication]: "2016/12/31"[Date - Publication])))) NOT short of breath[Title/Abstract]) NOT shortness of breath[Title/Abstract]) NOT breath sounds[Title/Abstract]) NOT gastric emptying[Title/Abstract])) AND ((((((((((proton transfer reaction mass spectrometry[Title/Abstract]) OR PTR-MS[Title/Abstract]) OR proton transfer reaction time-of-flight mass spectrometry[Title/Abstract]) OR PTR-TOF[Title/Abstract]) OR SIFT-MS[Title/Abstract]) OR selected ion flow tube mass spectrometry[Title/Abstract]) OR ion mobility spectrometry[Title/Abstract]) OR ion mobility mass spectrometry[Title/Abstract]) OR IMS-MS[Title/Abstract]) OR IMS[Title/Abstract]) AND ("1995/01/01"[Date - Publication]: "2016/12/31"[Date - Publication])	303
18	GC–MS	Exhaled Breath + VOCs and Headspace + Biological Media + Restrictions + GC–MS	((((((((((((((volatile organic compound[Title/Abstract]) OR VOC[Title/Abstract]) OR headspace[Title/Abstract]) AND ("1995/01/01"[Date - Publication]: "2016/12/31"[Date - Publication]))) AND ((((((((((blood[Title/Abstract]) OR urine[Title/Abstract]) OR feces[Title/Abstract]) OR faeces[Title/Abstract]) OR milk[Title/Abstract]) OR cell line[Title/Abstract]) OR cell culture[Title/Abstract]) OR bacteria[Title/Abstract]) OR saliva[Title/Abstract]) AND ("1995/01/01"[Date - Publication]: "2016/12/31"[Date - Publication])))) OR (((breath[Title/Abstract]) OR exhaled air[Title/Abstract]) AND ("1995/01/01"[Date - Publication]: "2016/12/31"[Date - Publication])))) NOT short of breath[Title/Abstract]) NOT shortness of breath[Title/Abstract]) NOT breath sounds[Title/Abstract]) NOT gastric emptying[Title/Abstract])) AND ((((((((((((((((((((gas chromatography-mass spectrometry[Title/Abstract]) OR GC–MS[Title/Abstract]) OR GC/MS[Title/Abstract]) OR GC–MS/MS[Title/Abstract]) OR GCxGC[Title/Abstract]) OR GCxGC-MS[Title/Abstract]) OR gas chromatography time-of-flight mass spectrometry[Title/Abstract]) OR GC-TOF-MS[Title/Abstract]) OR GC-TOF[Title/Abstract]) OR (gas chromatography[Title/Abstract] AND mass spectrometry[Title/Abstract])) OR (automated thermal desorption[Title/Abstract] AND gas chromatography[Title/Abstract])) OR ATD-GC/MS[Title/Abstract]) OR (ATD[Title/Abstract] AND GC–MS[Title/Abstract])) OR (orbitrap[Title/Abstract] AND GC[Title/Abstract])) OR (orbitrap[Title/Abstract] AND gas chromatography[Title/Abstract])) OR (QExactive[Title/Abstract] AND GC[Title/Abstract])) OR (QExactive[Title/Abstract] AND gas chromatography[Title/Abstract])) OR (ion trap[Title/Abstract] AND GC[Title/Abstract])) OR (ion trap[Title/Abstract] AND gas chromatography[Title/Abstract])) AND ("1995/01/01"[Date - Publication]: "2016/12/31"[Date - Publication]))	831
19	LC-MS	Exhaled Breath + VOCs and Headspace + Biological Media + Restrictions + LC-MS	((((((((((((volatile organic compound[Title/Abstract]) OR VOC[Title/Abstract]) OR headspace[Title/Abstract]) AND ("1995/01/01"[Date - Publication]: "2016/12/31"[Date - Publication]))) AND ((((((((((blood[Title/Abstract]) OR urine[Title/Abstract]) OR feces[Title/Abstract]) OR faeces[Title/Abstract]) OR milk[Title/Abstract]) OR cell line[Title/Abstract]) OR cell culture[Title/Abstract]) OR bacteria[Title/Abstract]) OR saliva[Title/Abstract]) AND ("1995/01/01"[Date - Publication]: "2016/12/31"[Date - Publication])))) OR (((breath[Title/Abstract]) OR exhaled air[Title/Abstract]) AND ("1995/01/01"[Date - Publication]: "2016/12/31"[Date - Publication])))) NOT short of breath[Title/Abstract]) NOT shortness of breath[Title/Abstract]) NOT breath sounds[Title/Abstract]) NOT gastric emptying[Title/Abstract] AND (((((((((((((((((((((((((((((liquid chromatography-mass spectrometry[Title/Abstract]) OR LC-MS[Title/Abstract]) OR LC/MS[Title/Abstract]) OR LC-MS/MS[Title/Abstract]) OR (liquid chromatography[Title/Abstract] AND mass spectrometry[Title/Abstract])) OR liquid chromatography time-of-flight mass spectrometry[Title/Abstract]) OR LC-TOF-MS[Title/Abstract]) OR ESI-MS[Title/Abstract]) OR electrospray ionization mass spectrometry[Title/Abstract]) OR high performance liquid chromatography-mass spectrometry[Title/Abstract]) OR HPLC-MS[Title/Abstract]) OR ultrahigh performance liquid chromatography-mass spectrometry[Title/Abstract]) OR UHPLC-MS[Title/Abstract]) OR ultrahigh performance liquid chromatography-high resolution mass spectrometry[Title/Abstract]) OR UHPLC-HRMS[Title/Abstract]) OR (UHPLC[Title/Abstract] AND MS[Title/Abstract])) OR (HPLC[Title/Abstract] AND MS[Title/Abstract])) OR (nanoelectrospray[Title/Abstract] AND mass spectrometry[Title/Abstract])) OR (ion trap[Title/Abstract] AND LC[Title/Abstract])) OR (ion trap[Title/Abstract] AND liquid chromatography[Title/Abstract])) OR (ion trap[Title/Abstract] AND LC[Title/Abstract])) OR (quadrupole[Title/Abstract] AND LC[Title/Abstract])) OR (quadrupole[Title/Abstract] AND liquid chromatography[Title/Abstract])) OR Q-TOF[Title/Abstract]) OR quadrupole-time-of-flight[Title/Abstract]) OR (orbitrap[Title/Abstract] AND LC[Title/Abstract])) OR (orbitrap[Title/Abstract] AND liquid chromatography[Title/Abstract])) OR (QExactive[Title/Abstract] AND LC[Title/Abstract])) OR (QExactive[Title/Abstract] AND liquid chromatography[Title/Abstract])) AND ("1995/01/01"[Date - Publication]: "2016/12/31"[Date - Publication])	187
20	Alcohol	Exhaled Breath 1960 + Alcohol	(((((((((breath[Title/Abstract]) OR exhaled air[Title/Abstract]) AND ("1960/01/01"[Date - Publication]: "2016/12/31"[Date - Publication]))) NOT shortness of breath[Title/Abstract]) NOT short of breath[Title/Abstract]) NOT breath sounds[Title/Abstract]) NOT gastric emptying[Title/Abstract])) AND (((alcohol[Title/Abstract]) OR ethanol[Title/Abstract]) AND ("1960/01/01"[Date - Publication]: "2016/12/31"[Date - Publication]))	1825
21	Sensors	Exhaled Breath + VOCs and Headspace + Biological Media + Restrictions + Sensors	((((((((((((((volatile organic compound[Title/Abstract]) OR VOC[Title/Abstract]) OR headspace[Title/Abstract]) AND ("1995/01/01"[Date - Publication]: "2016/12/31"[Date - Publication]))) AND ((((((((((blood[Title/Abstract]) OR urine[Title/Abstract]) OR feces[Title/Abstract]) OR faeces[Title/Abstract]) OR milk[Title/Abstract]) OR cell line[Title/Abstract]) OR cell culture[Title/Abstract]) OR bacteria[Title/Abstract]) OR saliva[Title/Abstract]) AND ("1995/01/01"[Date - Publication]: "2016/12/31"[Date - Publication])))) OR (((breath[Title/Abstract]) OR exhaled air[Title/Abstract]) AND ("1995/01/01"[Date - Publication]: "2016/12/31"[Date - Publication])))) NOT short of breath[Title/Abstract]) NOT shortness of breath[Title/Abstract]) NOT breath sounds[Title/Abstract]) NOT gastric emptying[Title/Abstract])) AND ((((((((((((((((electronic nose[Title/Abstract]) OR enose[Title/Abstract]) OR e-nose[Title/Abstract]) OR quantum cascade laser[Title/Abstract]) OR QCL[Title/Abstract]) OR fourier transform infrared spectroscopy[Title/Abstract]) OR FTIR[Title/Abstract]) OR non dispersive infrared sensor[Title/Abstract]) OR NDIR[Title/Abstract]) OR chemical array[Title/Abstract]) OR mid-infrared sensor[Title/Abstract]) OR sensor[Title/Abstract]) OR laser spectroscopy[Title/Abstract]) OR diode[Title/Abstract]) OR photoacoustic[Title/Abstract]) AND ("1995/01/01"[Date - Publication]: "2016/12/31"[Date - Publication]))	565

Table 4, Table 5, and 6 contain the number of breath research manuscripts published each year in the different topic areas. Table 4, Table 5, Table 6, and 6 correspond to the search strings listed in Table 1, Table 2, Table 3, respectively. Table 4, Table 5 contain the numbers of papers retrieved using the search string builders, while Table 6 contains the number of publications for each category in the final developed search strings for each breath research topic. Appendix A contains the lists of papers curated in each category using the final search strings found in Table 6. The manuscripts listed in Appendix A were utilized for review in Wallace et al. 2018. [1] The titles, author lists, journal names, publication dates, PMID numbers, and EntrezUID numbers are included in the Appendix A.

**Table 4:** Publications Per Year for Level 1 Searches. This table shows the breakdown of how many papers were published per year in each of the different searches from the level 1 search string builders shown in Table 1. Additional years are included for alcohol because this category was searched from 1960–2016. The total number of papers published over the entire timeframe for each category is listed at the bottom.

Year	Exhaled Breath (1)	VOCs (2)	Biological Media and Headspace (3)	Aerosols (4)	Condensate (5)	Direct MS (6)	GC–MS (7)	LC-MS (8)	Sensors (9)	Alcohol (10) (1995- 2016)	Year	Alcohol (10) (1960–1981)	Year	Alcohol (10) (1982–1994)
1995	773	67	47,809	3854	42	31	653	549	1014	487	**1960**	27	**1982**	236
1996	839	99	49,424	4113	69	32	779	682	1243	511	**1961**	28	**1983**	342
1997	861	92	49,907	4102	37	36	798	800	1364	462	**1962**	43	**1984**	363
1998	989	104	51,496	4235	59	51	832	900	1510	508	**1963**	55	**1985**	368
1999	1050	200	52,616	4941	53	64	977	1124	1611	568	**1964**	61	**1986**	375
2000	1043	209	54,763	5769	112	58	1150	1448	1980	576	**1965**	27	**1987**	425
2001	1035	288	55,745	6173	155	91	1313	1668	2222	610	**1966**	8	**1988**	404
2002	1044	303	56,804	6558	183	79	1387	1936	2508	636	**1967**	14	**1989**	430
2003	1107	331	59,561	6873	185	111	1429	2313	2651	593	**1968**	7	**1990**	444
2004	1145	385	61,578	8147	186	99	1530	2882	3518	665	**1969**	5	**1991**	477
2005	1274	371	64,445	9292	211	147	1642	3431	3905	682	**1970**	22	**1992**	447
2006	1257	388	66,209	9861	237	169	1727	3689	4297	616	**1971**	9	**1993**	494
2007	1231	499	69,320	10,020	230	194	1982	4387	4906	631	**1972**	16	**1994**	509
2008	1281	524	72,359	11,157	306	216	1976	4689	5613	723	**1973**	24		
2009	1341	485	74,931	11,857	266	259	2153	5182	6475	691	**1974**	22		
2010	1406	521	79,792	12,776	273	302	2390	5552	7111	763	**1975**	145		
2011	1456	583	85,508	13,421	264	382	2517	6244	7944	790	**1976**	134		
2012	1650	658	94,022	14,489	321	438	2804	7069	8785	810	**1977**	121		
2013	1795	723	100,248	15,553	290	500	2855	7800	10,241	986	**1978**	156		
2014	1805	854	105,204	16,746	324	551	3046	8288	11,443	1047	**1979**	160		
2015	1877	821	109,498	17,256	331	582	3083	8917	12,187	925	**1980**	206		
2016	1924	899	110,358	17,996	323	592	3196	9214	13,234	948	**1981**	213		
Total	28,183	9404	1,571,597	215,189	4457	4984	40,219	88,764	115,762	15,228		1503		5314
Alcohol Total														22045

**Table 5:** Publications Per Year for Level 2 Searches. This table shows the number of publications found per year using the PubMed search strings from level 2, which are listed in Table 2. The total number of papers published over the entire timeframe for each category is listed at the bottom.

Year	VOC + Biological Media (11)	Exhaled Breath + Restrictions (12)	Exhaled Breath + VOC + Biological Media (13)	Exhaled Breath + VOC + Biological Media + Restrictions (14)
1995	21	651	794	672
1996	32	706	864	731
1997	26	713	884	736
1998	26	829	1015	855
1999	52	882	1100	931
2000	41	871	1083	911
2001	65	843	1096	904
2002	63	843	1103	902
2003	56	900	1155	948
2004	64	921	1207	983
2005	67	1009	1336	1071
2006	57	1007	1310	1060
2007	67	966	1297	1032
2008	66	981	1344	1044
2009	74	1000	1408	1067
2010	62	1045	1464	1103
2011	59	1063	1510	1117
2012	84	1180	1727	1257
2013	94	1318	1877	1400
2014	105	1344	1896	1435
2015	96	1374	1961	1458
2016	124	1397	2036	1509
Total	1401	21,843	29,467	23,126

**Table 6:** Publications Per Year for Level 3 Searches. This table shows the number of papers published each year in the selected topic areas. These papers were curated using the search strings listed in Table 3. The total number of papers published in each category as well as the alcohol total for all of the columns are shown at the bottom.

Year	Exhaled Breath Aerosols (15)	Exhaled Breath Condensate (16)	Direct-MS (17)	GC–MS (18)	LC-MS (19)	Sensors (20)	Alcohol (21) (1995–2016)	Year	Alcohol (21) (1960–1982)	Year	Alcohol (21) (1983–1994)
1995	11	2	1	5	1	8	52	**1960**	0	**1983**	17
1996	15	6	0	11	2	9	54	**1961**	0	**1984**	32
1997	15	5	2	9	0	6	41	**1962**	1	**1985**	28
1998	16	9	0	9	1	4	44	**1963**	0	**1986**	23
1999	18	10	4	15	1	5	57	**1964**	3	**1987**	37
2000	21	13	3	21	2	6	48	**1965**	4	**1988**	33
2001	20	19	6	29	1	6	35	**1966**	2	**1989**	47
2002	25	32	4	26	5	13	46	**1967**	1	**1990**	45
2003	17	55	9	20	5	9	47	**1968**	1	**1991**	52
2004	16	52	3	31	5	16	45	**1969**	2	**1992**	59
2005	27	66	10	28	7	15	44	**1970**	1	**1993**	47
2006	22	82	15	28	9	11	54	**1971**	9	**1994**	32
2007	25	70	10	40	10	19	62	**1972**	5		
2008	29	120	15	46	5	25	50	**1974**	7		
2009	24	85	24	51	11	25	50	**1975**	5		
2010	25	101	19	48	13	20	57	**1976**	5		
2011	24	90	25	42	17	35	62	**1977**	11		
2012	41	123	26	69	17	39	62	**1978**	12		
2013	34	92	27	70	18	65	91	**1979**	3		
2014	36	93	31	78	19	67	87	**1980**	14		
2015	33	97	35	77	17	77	81	**1981**	7		
2016	40	90	34	66	16	77	80	**1982**	13		
Total	534	1312	303	819	182	557	1249		106		452
Alcohol Total											1807

## 2 Experimental design, materials, and methods

Publications reporting breath research data in the topic areas EBA, EBC, Direct MS, GC–MS, LC-MS, alcohol, and sensors were compiled and organized. The PubMed Advanced Search Builder available from NCBI was utilized to curate papers (www.ncbi.nlm.nih.gov/pubmed/advanced). The PubMed database was accessed in July 2017. All keyword searches were limited to the Title/Abstract of the paper to ensure that each topic area was the main focus of the article. Searches were date restricted from 01/01/1995 to 12/31/2016 for all categories except alcohol, which was restricted from 01/01/1960 to 12/31/2016. The year range from 1995–2016 was selected because breath research became more prevalent in 1995 in many of the selected topics. For breath alcohol research, a longer time span was implemented because alcohol research began earlier than many of the other types of breath research due to the invention of the breath alcohol samplers (e.g., breathalyzers) [2], [3].

The PubMed search strings were developed in three levels. In the first level, shown in Table 1, short search strings for individual aspects of the final topics were created. These topics included “exhaled breath,” “VOCs and headspace,” “biological media,” “aerosols,” “condensate,” “Direct MS,” “GC–MS,” “LC-MS,” “alcohol,” and “sensors.” Manuscripts in the assigned timeframes were retrieved for these individual topics in the level 1 searches. These individual short search strings are later combined with one another to retrieve papers related to both exhaled breath AND the seven selected topics (i.e., EBA, EBC, Direct MS, GC–MS, LC-MS, alcohol, and sensors). Each search string was assigned a search number (column 1), which is used to differentiate between the developed search strings in the method. Where applicable, “OR” statements were included to provide additional options for synonyms, abbreviations, and acronyms that may be utilized in the literature. While “exhaled breath,” “VOCs and headspace,” and “biological media” are not included as final categories themselves, development of these search strings was necessary to provide the framework for ensuring that the papers retrieved in each of the seven final categories were related to exhaled breath research or the analysis of VOCs from biological media/headspace. For the instrumental search strings, “Direct MS,” “GC–MS,” “LC-MS,” and “sensors,” different instrument names were included in the search string to increase the possibility of retrieving all relevant papers. These lists are not exhaustive and could be modified in future studies to include additional parameters depending on research interests. The numbers of publications returned for each of these individual search string builders from level 1 are provided in Table 1.

Table 2 contains combinations of the search string builders developed in Table 1. The search string builders from Table 1 were combined using “AND” statements between them to narrow down the subject area of searches 11–14. For example, in search 11, “VOCs and headspace (2)” was combined with “biological media (3)” to curate papers regarding the study of VOCs emitted from biological media, such as blood, urine, or cell culture. Additionally, some restrictions were added to searches 12 and 14 to eliminate papers that were unrelated to exhaled breath research. The abstracts of the papers curated using the search strings listed in Table 1 were scanned to find keywords that indicated the article did not include the analysis of exhaled breath. These words and phrases that were restricted tended to describe the health state of patients in medical journals that did not involve the analysis of exhaled breath. To eliminate the return of these types of papers in the searches, “NOT” statements were added to the search strings to (Table 2). These “NOT” statements included “shortness of breath,” “short of breath,” “breath sounds,” and “gastric emptying.” While this list is not exhaustive, this step was included to filter out papers that were likely not related to the topic areas, and thus manual elimination and review of individual papers to see if they fit within the topic areas was not performed after this step.

The level 3 search strings for the final topic areas EBA, EBC, Direct MS, GC–MS, LC-MS, alcohol, and sensors can be found in Table 3. These final search strings are a combination of the search string builders shown in Table 1, Table 2. The EBA, EBC, and alcohol searches only included the search string for “Exhaled Breath + Restrictions (12),” as the searches for these topic areas were intended to only retrieve papers regarding sampling from humans. Searches for GC–MS, LC-MS, Direct-MS, and sensors were built upon the search string “Exhaled Breath + VOCs and Headspace + Biological Media + Restrictions (14),” which included blood, urine, feces, faeces, milk, cell line, cell culture, bacteria, or saliva to retrieve articles related to sampling cellular and biological headspace in addition to exhaled breath, EBA, and EBC.

The number of papers retrieved per year using each of the different search strings listed in Table 1, Table 2, Table 3 was also documented. This information was obtained from the PubMed website. After each search, a graph showing the “Results by year” appeared automatically with the PubMed search results. To obtain the output of this information, the “Download CSV” link below each graph was selected, and the.csv file was opened in Microsoft Excel. The data were sorted by year from 1995–2017, and only the data for 1995–2016 were included in the tables, graphs, and final assessments of publication numbers. For determination of article publication year, the final published date appearing in the journal was utilized instead of the online (EPub) date.

Table 4 contains the categories and publication numbers for the search strings listed in Table 1 (i.e., “exhaled breath (1),” “VOCs,” “biological media and headspace (3),” “aerosols (4),” “condensate (5),” “Direct MS (6),” “GC–MS (7),” “LC-MS (8),” “sensors (9),” and “alcohol (10)).” The data for alcohol is shown in three different columns to cover the years from 1960–2016, while the rest of the categories are shown from 1995–2016. Similarly, the data for the publications per year for the search strings shown in Table 2 are listed in Table 5, which contains the combined search string builders “VOC + biological media (11),” “exhaled Breath + restrictions (12),” “exhaled breath + VOC + biological media (13),” and “exhaled breath + VOC + biological media + restrictions (14)” from 1995–2016. The total number of publications for each category is shown at the bottom of each table (the sum from 1995–2016). The alcohol total is included as the last row and is the sum of the three individual alcohol columns for all manuscripts published from 1960–2016.

The numbers of papers published per year for the level 3 search strings are shown in Table 6. These numbers were curated using the final search strings listed in Table 3. These topics include exhaled breath and instrumentation or alcohol, and were searched from 1995–2016 for all topics except alcohol (searched from 1960–2016; shown in three columns). The total number of publications for each topic over the time period is included at the bottom of the table, and the total for alcohol (the sum of the three alcohol columns) is included as the “alcohol total.”

The final lists of all publications from each category that were curated using the final search strings in Table 3 can be found in Appendix A. These publication lists were downloaded from the PubMed website after each search was completed. The “send to” link at the top of the web page was selected, and the destination “File” was chosen. The format was selected as “CSV” and the articles were sorted by “Most Recent.” The.csv files were opened in Microsoft Excel. The spreadsheets were filtered to remove any articles that were published in 2017. Appendix A contains the titles, author lists, journal names, publication dates, PMID numbers, and EntrezUID numbers for the topics exhaled breath aerosols, exhaled breath condensate, Direct MS, GC–MS, LC-MS, alcohol, and sensors, listed in Table 3 and 6. These data were utilized to discern publication trends and review the history of breath research literature in Wallace et al. 2018. [1].
