# Supplementary material for: Dataset of breath research manuscripts curated using PubMed search strings from 1995–2016
Source: Data Brief. 2018 May 2;18:1711–24. doi: 10.1016/j.dib.2018.04.063 (PMC5998180; doi:10.1016/j.dib.2018.04.063)
Supplement: Supplementary file 1 — Supplementary material [file mmc1.docx]

**Conflict of interest disclosure**

The authors declare no competing financial interest.
