# Supplementary material for: Dataset of breath research manuscripts curated using PubMed search strings from 1995–2016
Source: Data Brief. 2018 May 2;18:1711–24. doi: 10.1016/j.dib.2018.04.063 (PMC5998180; doi:10.1016/j.dib.2018.04.063)
Supplement: Supplementary file 2 — Supplementary material [file mmc2.doc]

# Five Chances in Evolution

**Grant Ramsey, Charles H. Pence (eds.), *Chance in Evolution*, University of Chicago Press, Chicago (2016). Pp. 359, Price $45.00 cloth, ISBN: 978-0-226-40188-1**

By Carlos Mariscal^1,2,3*^ & Alexander Lerner^1^

University of Nevada, Reno

^1^ Department of Philosophy

^2^ Ecology, Evolution, and Conservation Biology Program

^3^ Integrative Neuroscience Program

^*^ Corresponding author

Carlos Mariscal's E-mail address is [carlos@unr.edu](mailto:carlos@unr.edu)

## Chance in Evolution

Among the central issues in philosophy of biology is the role of chance in evolution. Evolutionary theory relies on several chance concepts, including ‘random’ drift, which is where population outcomes differ by ‘chance’ from what is expected by natural selection. But the nature of drift is hotly debated: some have argued it is merely a place-holder for our own ignorance, others have argued it is not a force in evolution, while still others have argued it is not a distinct process in biology at all. Understanding chance in evolution required the development of the field of statistics and set the stage for a conflict between scientists and those who argued that the design apparent in nature was incompatible with ‘mere chance.’ These authors were often impressed that camera-type eyes have evolved a half-dozen times, a phenomenon that also poses questions for practicing biologists who wrestle with whether this implies evolutionary forms are limited. From these debates, we can distinguish at least five issues raised by the concept of chance in biology:

### i. whether chance in biology is objective, of the same sort as those we see in quantum theory, or whether probabilities merely reflect our own epistemic barriers (e.g., Horan 1994, Brandon & Carson 1996, Graves et al. 1999);

### ii. whether evolutionary processes are forces or merely a statistical summary of underlying processes (e.g., Walsh et al. 2002, Stephens 2004, Walsh et al. 2017);

### iii. whether indiscriminate chancy biological processes are separate from selective processes or whether both are aspects of a single process (e.g., Beatty 1984, Millstein 2002, Brandon 2005);

### iv. whether chance is compatible with teleology or not (e.g., Ruse 1996, 2003).

### v. whether evolution is likely to produce repeat outcomes given radically distinct starting points or whether current features owe their occurrence to the chance events in a lineage’s contingent history (e.g., Gould 1990, Conway Morris 1998, Powell & Mariscal 2015);

Chance and related concepts are used in each debate in different ways, so even experts may be unclear as to how these debates intersect, if at all. Each is often posed as an exclusive dichotomy, exhaustive of all possibilities within the issue. Each question is also sometimes taken to be central to understanding biology. Debates ***ii*** and ***v***, in particular, are still actively debated in academic circles, as is ***iv*** across academia and the public sphere.

In this article, we describe each of these debates for audiences who may have a passing interest but are not actively versed in the issues. The historical roots and conceptual ordering for these debates for some of these debates is ambiguous, so we have organized our discussion in the order they arise within the new edited anthology *Chance in Evolution*, Grant Ramsey (KU-Leuven) and Charles Pence (Louisiana State University), which explores such debates side-by-side with the myriad issues raised within. Both editors are well known in these debates, especially with respect to ***ii*** and ***iii*** above (e.g., Brandon & Ramsey 2007, Ramsey 2013a,b, Pence & Ramsey 2013, Pence 2015, Pence 2017).[[1]](#footnote-2)

## Epistemic vs. Ontological Chanciness

In the mid-1990s, a debate raged in the philosophy of biology community as to the causes of genetic drift and other chancy biological processes. In one view, the unpredictability of such processes is due to our own epistemic limitations–the chance in biology is subjective, not objective (e.g., Horan 1994, Rosenberg 1994, Graves et al. 1999). In other words, populations drift because of deterministic factors we are currently unable to measure, but an ideal scientist could, in principle, perfectly predict the drift of any population. The alternative view is that drift is not an epistemic limitation, like throwing dice in a casino, but it is truly stochastic in a way similar to quantum mechanics (Brandon & Carson 1996). In the early 2000s, Leslie Graves and Barbara Horan left academic philosophy, while Alex Rosenberg abandoned the view that drift was a mere epistemic limitation (Rosenberg 2001, Bouchard & Rosenberg 2004, Rosenberg & Bouchard 2005). The debate has now largely disappeared from philosophy journals.

The first chapter of this anthology takes us to the historical roots of this debate. David Depew writes a broad overview from the ancients to the Modern Synthesis. Depew covers Aristotle and Empedocles on the generation of species and their seemingly teleological natures, Immanuel Kant explicating the nature of species disposition, Charles Darwin and Asa Gray's discussions on the nature of species, and even touches on the probability revolution and ongoing research by modern thinkers. To understand their differing theories, Depew assesses the use of the term *chance* as its use varies from thinker to thinker. Sometimes chance is equivalent to *contingency,* as in Aristotle's two versions of luck, whereas other times it is closer to *stochasticity*. Darwin includes the idea of random variation in his nascent theory of natural selection, yet considers that chance may simply be the incomplete understanding of the science before us. Mutability is another avenue by which chance is examined, calling back through history to Aristotle’s epigenesis, and leaning heavily on contemporary research in the field of genetics. Depew’s contribution to the work explains that chance has always been present in the theory of evolution in its varied iterations. For Depew, if the Darwinian revolution was truly revolutionary, it was not revolutionary in the sense that Darwin broke with the ancients, but rather that he broke with certain beliefs that became common in the century before him. Darwin had a hard time finding a middle ground between random chance and determinism, one in which a concept of contingency might have helped (see section 4). Depew’s article may be a worthwhile entry point for historically inclined scholars.

On a very different topic, Francesca Merlin pens a chapter discussing mutation and how it is observed in science. Merlin argues weak randomness is a more realistic way of viewing the randomness associated with mutation. *Weak randomness* is any stochastic process that is either a discriminate sampling process or variant over time. Research shows some pattern to changing probabilities of mutation, so mutation exhibits weak randomness (Ninio 1991, Drake et al. 2005, Drake 2007). The author looks to future research to improve understanding of mutability biases.

## Statisticalism vs. Causalism

Evolution is often described in causal terms: selective pressures, migration, and mutation all have an *effect* on biological populations. Some authors view these causes as biological ‘forces,’ analogous to forces in physics (Bouchard & Rosenberg 2004, Stephens 2004, Reisman & Forber 2005, Shapiro & Sober 2007, Brandon & Ramsey 2007, McShea & Brandon 2010, Ramsey 2013b, Pence 2017). An alternative view, ‘statisticalism,’ holds that the parameters in evolutionary models explain, predict, and quantify changes in population structure, but do not describe *causes*. For these authors, true causes can only be said to occur locally, among the births and deaths of particular organisms, and our evolutionary explanations are mere statistical aggregates (Matthen & Ariew 2002, Ariew & Lewontin 2004, Ariew & Ernst 2009, Walsh 2010, Walsh et al. 2017). A recent review by Otsuka (2016) does an excellent job going through this debate and we encourage readers to read that work.

An interesting new addition to this debate in *Chance in Evolution* is in the second chapter, by Jonathan Hodge. Hodge gives a thorough overview of Darwin's developing theory and Darwin’s shift, over time, on his view on the force of contingency. Darwin begins with two premises: variations are due to chance and probabilities are causally related. He believed that chance variations were rare and bred out in populations, securing the place of certain fundamental traits. Chance then became simply a lack of understanding, and this would eventually lead Darwin to soft determinism. Darwin would reformulate his ideas, writing that both drift and natural selection were causal forces and causing an intellectual uproar with his acknowledgment of maladaptations, challenging the thinkers who had committed to intelligent design and theological explanations for change. Hodge weighs in on the ongoing argument between statisticalists and causalists, arguing that a historical reading favors the causalist perspective, albeit warning that the concepts of ‘forces’ and ‘laws’ have a checkered history in biology, as does linking ‘fitness’ with reproductive output. This serves as a specific example of a general point: philosophers should pay a keen eye to history: not only are debates shaped by their history, but so too is our conception of the issues. Hodge’s chapter does well in advancing the statisticalist/causalist debate, and should be read by anybody participating in that discussion.

## The Nature of Drift

One interesting consequence of the previous debate was a reconceptualization of drift. The traditional position was that drift was an unbiased sampling process or cause (Mills & Beatty 1979, Beatty 1984, Millstein 2002, Bouchard and Rosenberg 2004, Gildenhuys 2009). The new view, in opposition to the mainstream position, is that drift is merely the byproduct of a single process (namely the births and deaths of organisms (Walsh et al. 2002, Brandon 2005). There are alternative possibilities: Gildenhuys 2009 accepts both, while Ramsey 2013a views drift as an individual-level phenomenon.

In the book, several authors wrestle with how to understand drift in a way that can illuminate this debate. In one chapter, Anna Plutynski, Kenneth Vernon, Lukas Matthews, and Daniel Molter explore the myriad conceptions of chance used by each major evolutionary biologist throughout the modern synthesis. The authors argue for an appreciation of the lineage of work in the changing viewpoints in biological theory. Chance was recognized by modern synthesis authors as occurring within mutation, meiosis, small populations, and drift. Later synthesis authors would keep to a similar core of commitments, accepting that chance in mutation and recombination was inherent, although underplaying the role of drift as the synthesis ‘hardened’ (Gould 1983). The authors of this chapter acknowledge that synthesis authors all held a respect for some notion of ‘chance’ (in the sense of unpredictable) or ‘randomness’ (in the sense of equiprobable outcomes) within the theory of evolution. Most would also have viewed the debate between causalists and statisticalists as a false choice. This chapter would be particularly helpful in a course when discussing the Modern Synthesis, especially leading into one of these debates. Missing in the gap between Darwin and this chapter are the biometricians, Karl Pearson and Walter Weldon, who founded the field of statistics. Readers can see the great work of one of the editors for more on both (Pence 2011, 2015).

In a later chapter, Michael Strevens tackles the reference class problem for evolutionary biology. The reference class problem is how to assign a probability to an event when the event can factor into many reference classes, each of which can be given their own probabilities. The fat, old cat is both a fat cat and an old cat, each of which has a certain probability of dying, so which statistics should I use when estimating whether it will die this year? Strevens’ solution for biology is to treat three formal features of a system as determining their probabilities, and treat all others as making no difference. These are the *microconstancy*, which occurs when the space of initial conditions can be divided into small contiguous regions, the *strike ratio*, which is the proportion of each condition producing the same outcome, and the *smoothness*, which is when probabilities change only slowly across small intervals. So, if cats are likely to die in even years but not in odd years, the probability distribution of a cat dying will be different than if their probabilities of dying are constant. These conditions, Strevens argues, help resolve the reference class problem and ensure we focus on one single causal mechanism and not many disjunctive ones. It is unlikely to be the final word on the matter, but is an interesting addition to the discussion.

These chapters may not convince any partisans to leave their favorite understanding of drift, but they do add new layers of understanding to the debate for those of us on the sidelines.

## Chance vs. Teleology

The final debate we discuss is also, in a way, the first debate that troubled Darwin, seen most clearly through his discussions with Asa Gray. Here we consider twin challenges that faced Darwin’s *Origin* from religious and secular concerns about teleology. On the religious side, it seemed as if Darwinian evolution left no room for intelligent design. The chanciness of biological evolution seemed to go against an all-knowing, benevolent God. On the secular side, some have worried that there is an inherent tension in the accumulation of small chancy events producing what appears to be a design. This is often brought up by creationists as a reason to doubt Darwinian evolution, although most biologists don’t find the tension at all motivating.

Two chapters cover this topic. J. Matthew Ashley explains how Christian thinkers, specifically Charles Hodge and Cristoph Schönborn, regarded Darwin’s theory and its seeming abandonment of design in favor of randomness. The chapter makes a thorough examination of the claims of intelligent design and how theologians accepted and incorporated the theory of evolution while redefining *chance* as *divine providence*, *contingency* as *the will of God*, and *randomness* as *purpose*. Ashley shows in this chapter that research coming from religious backgrounds has contributed to the theory of evolution, and that, despite an uneasy history with the cultural departure from the teleological conception, it has become more inclusive, flexible, and able to collaborate with contemporary, secular theories.

Finally, Michael Ruse begins his chapter by pointing out that one of the many great realizations that Darwin would leave us as his legacy was the realization that we as a species are not the culmination of the great chain of being but just another species in the tree. Ruse points out Darwin’s reluctance to discuss humans during the creation of his theory, noting the effect this would have academically and theologically in his time. This has precipitated an interesting query: were we just the product of a biological arms race for intelligence, and is that as rare as we believe it to be? Ruse ends the chapter with the poignant observation that no philosopher would commit themselves to saying that humans were inevitable yet, just as few would say our rise was purely by chance.

## Convergence vs. Contingency

One long-running debate in evolutionary biology is the extent to which change is predictable and the extent to which it is dependent on initial conditions. Stephen J. Gould famously ignited the debate in *Wonderful Life* by claiming that if we ‘re-ran the tape of life,’ then the results would be completely different. Gould used epistemological, stochastic, and path-dependency arguments to defend his metaphor, and much ink has been spilled in its interpretation (e.g., Beatty 2006, Powell 2007, Turner 2011, Currie 2012, Powell & Mariscal 2015). We’ll consider the notion of ‘path dependency’ here to typify Gould’s argument, but point out that there is little consensus on Gould exegesis.

Much of this book can fit into this debate. Starting in chapter 8, the collective efforts of Thomas Lenormand, Luis-Miguel Chevin, and Thomas Bataillon seek to explain selectionist and mutationist views and how parallel evolution has benefited from emerging science in its ability to focus in on processes in action and not just outcomes. In their overview of genetics, the authors develop a strong commentary on the current use of stereotypes in understanding mutations and the current understanding of pleiotropy and magnitude in gene expression. The authors give a brief synopsis of the challenges of mutations, reductionism, and current modeling techniques while staying hopeful that continuing efforts may yield an underlying law with predictable consequences for species evolution.

Path dependence, the concept that the future depends on the choices of the past, is the backdrop of Eric Desjardins chapter which examines how to view the past and its effects on the current evolutionary stage of a species. He correctly identifies the difficulty inherent in understanding which past occurrences can be affective in nature and which are negligible. Using current lab work and studies on Sawbugs and Sprucebugs, Desjardins lays out a conversation about evolutionary constraints and how they can be useful in arguing path dependence. Genetic entrenchment is also a concept that is used to show that we can eliminate randomness to some degree when we have a greater understanding of how to walk back phenotypic and genotypic histories. The author concludes with an affirmation that it is not entirely possible to eliminate chance with a historical examination and allows that history, path dependence, and chance all have their place in our current models.

Citing convergence, limited niches, and limited phylogenetic answers to environmental stresses, author Zachary Blount acknowledges a strong argument for the deterministic nature of evolution. Using parallel replay experiments, historical difference experiments, and invoking the LTEE experiment the author notes that contingency cannot be ruled out entirely. Blount calls out to the interdisciplinary science of evolution, urging a collaborative effort to push the field of contingency and the notion of evolvability into the forum in hopes of giving credence to the idea that chance does have its place.

Betül Kaçar’s fascinating work focuses on harnessing the mechanical structure of contemporary cells to host ancient proteins in hopes of understanding how these antiquated proteins worked and evolved into more modern structures. Kaçar’s ongoing work may help show how evolutionary trajectory can be studied by current methods and we can begin, as scientists, to “rewind the tape” of protein evolution. Students and scientists alike may be inspired by this experimental approach to understanding the processes of chance and contingency, and we can see this work being referenced in a number of biology courses.

Finally, we turn to Douglas Erwin who recounts Stephen Gould's work with the Burgess Shale fossils and how his deterministic views changed, viewing selection and adaptation as over-glorified evolutionary pressures. Gould commits to the idea that things could have and likely would have turned out differently if given the chance. Contemporary work undermines Gould's conclusions, citing convergence as the main antagonist to his theories and defending a “developmental toolkit” theory that is endemic to each species' embryos. The text ends with an observation that we want patterns and seek evidence against contingency despite not being able to correctly define it. The author elaborates that contingency may not even be recognizable to us at this stage and that chance may play on a whole domain we have yet to recognize. The chapter concludes by affirming that chance cannot yet be written out of the story of evolution.

## Conclusion

We have done a whirlwind tour of chance concepts in biology, guided by Ramsey & Pence’s *Chance in Evolution*. We’ve presented five separate ways people have disagreed about the role of chance in biology. This list is not exhaustive; experts may further subdivide each category and new debates may yet emerge. These debates illustrate how so much trouble can be raised from such a seemingly simple concept like ‘chance.’

Generalist philosophers of science might wish to be aware of the myriad issues when applying chance concepts to biology or other special sciences. The abandonment of an epistemic understanding of chance in philosophy of biology may be of interest to both general philosophers of science and biologists (Fleming 2017). The rise of philosophy of biology in the 1970s was spurred by realization that biology greatly differed from physics in its theories, models, explanations, and assumptions. It was only in the exploration of these issues that philosophers of science became aware of the diversity of concepts that may underlie even the most technical terms. It is probably wise for even generalists to check in on the philosophies of special sciences from time to time to see how certain issues play out.

For updated work on each of these debates, we suggest this book. It builds a narrative arch that illustrates how this area of research benefits from collaborations across disciplines as diverse as science, history, and philosophy. From experience, we can confirm a graduate-level special topics in philosophy of biology course could easily be based on this book, supplemented by some of the more famous papers in each of the five debates described above. It would also be an extremely fruitful resource to mine in history and biology courses, especially on Darwinian thought, genetics, or evolutionary theory. This book fills the gap left by undergraduate and graduate courses that avoid historical or philosophical presentations of biology. It is essential reading for practitioners in any of the debates we’ve discussed, and just a good book to read or add to your bookshelf.

**References**

Ariew, A., & Ernst, Z. (2009). What fitness can’t be. *Erkenntnis*, *71*(3), 289.

Ariew, A., & Lewontin, R. C. (2004). The confusions of fitness. *The British Journal for the Philosophy of Science*, *55*(2), 347-363.

Beatty, J. (1984). Chance and natural selection. *Philosophy of science*, *51*(2), 183-211.

Bouchard, F., & Rosenberg, A. (2004). Fitness, probability and the principles of natural selection. *The British Journal for the Philosophy of Science*, *55*(4), 693-712.

Brandon, R. N. (2005). The difference between selection and drift: A reply to Millstein. *Biology and Philosophy*, *20*(1), 153-170.

Brandon, R. N., & Carson, S. (1996). The indeterministic character of evolutionary theory: no" no hidden variables proof" but no room for determinism either. *Philosophy of Science*, *63*(3), 315-337.

Brandon, R. N., & Ramsey, G. (2007). *What's wrong with the emergentist statistical interpretation of natural selection and random drift?* In: Hull DL, Ruse M (eds) The cambridge companion to the philosophy of biology. Cambridge University Press, Cambridge, pp 66–84.

Conway Morris, S. (1998). *The crucible of creation: the Burgess Shale and the rise of animals*. Peterson's.

Currie, A. (2012). Convergence as evidence. *The British Journal for the Philosophy of Science*, *64*(4), 763-786.

Drake, J. W. (2007). Too many mutants with multiple mutations. *Critical reviews in biochemistry and molecular biology*, *42*(4), 247-258.

Drake, J. W., Bebenek, A., Kissling, G. E., & Peddada, S. (2005). Clusters of mutations from transient hypermutability. *Proceedings of the National Academy of Sciences of the United States of America*, *102*(36), 12849-12854.

Fleming, L. (2017). Two Dogmas of Biology. *Philosophy, Theory, and Practice in Biology*, *9(2). DOI: http://dx.doi.org/10.3998/ptb.6959004.0009.002*.

Gildenhuys, P. (2009). An explication of the causal dimension of drift. *The British Journal for the Philosophy of Science*, *60*(3), 521-555.

Gould, S. J. (1983). The Hardening of the Modern Synthesis. In: Dimensions of Darwinism, ed. Marjorie Grene. Cambridge University Press, 71-93.

— (1990). *Wonderful life: the Burgess Shale and the nature of history*. WW Norton & Company.

Graves, L., Horan, B. L., & Rosenberg, A. (1999). Is indeterminism the source of the statistical character of evolutionary theory?. *Philosophy of Science*, *66*(1), 140-157.

Horan, B. L. (1994). The statistical character of evolutionary theory. *Philosophy of Science*, *61*(1), 76-95.

Matthen, M., & Ariew, A. (2002). Two ways of thinking about fitness and natural selection. *The Journal of Philosophy*, *99*(2), 55-83.

McShea, D. W., & Brandon, R. N. (2010). *Biology's first law: the tendency for diversity and complexity to increase in evolutionary systems*. University of Chicago Press.

Mills, S. K., & Beatty, J. H. (1979). The propensity interpretation of fitness. *Philosophy of Science*, *46*(2), 263-286.

Millstein, R. L. (2002). Are random drift and natural selection conceptually distinct?. *Biology and Philosophy*, *17*(1), 33-53.

Ninio, J. (1991). Transient mutators: a semiquantitative analysis of the influence of translation and transcription errors on mutation rates. *Genetics*, *129*(3), 957-962.

Otsuka, J. (2016). A critical review of the statisticalist debate. *Biology & Philosophy*, *31*(4), 459-482.

Pence, C. H., & Ramsey, G. (2013). A new foundation for the propensity interpretation of fitness. *The British Journal for the Philosophy of Science*, *64*(4), 851-881.

Pence, C. H. (2011). “Describing our whole experience”: The statistical philosophies of WFR Weldon and Karl Pearson. *Studies in History and Philosophy of Science Part C: Studies in History and Philosophy of Biological and Biomedical Sciences*, *42*(4), 475-485.

— (2015). The early history of chance in evolution. *Studies in History and Philosophy of Science Part A*, *50*, 48-58.

— (2017). Is genetic drift a force? *Synthese*, *194*(6), 1967-1988.

Powell, R., & Mariscal, C. (2015). Convergent evolution as natural experiment: the tape of life reconsidered. *Interface focus*, *5*(6), 20150040.

Ramsey, G. (2013a). Driftability. *Synthese*, *190*(17), 3909-3928.

—(2013b). Organisms, traits, and population subdivisions: Two arguments against the causal conception of fitness? *The British Journal for the Philosophy of Science*, *64*(3), 589-608.

Reisman, K., & Forber, P. (2005). Manipulation and the Causes of Evolution. *Philosophy of Science*, *72*(5), 1113-1123.

Rosenberg, A. (1994). *Instrumental biology, or the disunity of science*. University of Chicago Press.

— (2001). Discussion note: Indeterminism, probability, and randomness in evolutionary theory. Philosophy of Science, 68(4), 536-544.

Rosenberg, A. & Bouchard, F. (2005). Matthen and Ariew's obituary for fitness: reports of its death have been greatly exaggerated. *Biology and Philosophy*, *20*(2), 343-353.

Ruse, M. (1996). *Monad to man: the concept of progress in evolutionary biology*. Harvard University Press.

— (2003). *Darwin and design: does evolution have a purpose?*. Harvard University Press.

Shapiro L. A. & Sober, E. (2007). Epiphenomenalism—the dos and the don’ts. In: Wolters G, Machamer P (eds) *Thinking about causes: from Greek philosophy to modern physics*. University of Pittsburgh Press, Pittsburgh

Stephens, C. (2004). Selection, drift, and the “forces” of evolution. *Philosophy of Science*, *71*(4), 550-570.

Turner, D. D. (2011). Gould’s replay revisited. *Biology & Philosophy*, *26*(1), 65-79.

Walsh, D. M. (2010). Not a sure thing: Fitness, probability, and causation. *Philosophy of Science*, *77*(2), 147-171.

Walsh, D. M., Lewens, T., & Ariew, A. (2002). The trials of life: Natural selection and random drift. *Philosophy of Science*, *69*(3), 429-446.

Walsh, D. M., Ariew, A., & Matthen, M. (2017). Four Pillars of Statisticalism. *Philosophy & Theory in Biology*, *9*.

1. Full disclosure, Ramsey shared academic advisors with **CM**. [↑](#footnote-ref-2)
