# Supplementary material for: Dataset of breath research manuscripts curated using PubMed search strings from 1995–2016
Source: Data Brief. 2018 May 2;18:1711–24. doi: 10.1016/j.dib.2018.04.063 (PMC5998180; doi:10.1016/j.dib.2018.04.063)
Supplement: Supplementary file 3 — Supplementary material [file mmc3.docx]

**"What (good) is Cultural History for History of Science today?**

**Perspectives, Challenges, Concerns"**

Stéphane Van Damme

European University Institute, Stephane.VanDamme@eui.eu

Bernard Lightman (ed.), *A Companion to the history of science*. Chichester: John Wiley & Sons and Blackwell, 2016. xvi+601 pp. ISBN-13: 978-1-118-62077-9. £120.00 (hardback)

Given the unprecedented speed and depth of the transformation apparent in current developments and research trends in the history of science in the last three decades, the moment has undoubtedly come to map them anew. It can be no coincidence that a similar ambition has given rise to several such undertakings in recent years if we think of the volumes of the *Cambridge History of Science* edited by Ronald Numbers and David Lindberg or the recent *Histoire des sciences et des savoirs*^[[1]](#footnote-1)^. However, this *Companion* chooses to reject the chronological and geographical organisation adopted by those volumes; it stands out as original for providing an entirely thematic overview of thirty years of research. Encyclopaedic in scope, the book has deep sociological, spatial, and material roots. It charts research themes in detail with contributions from the finest scholars in the field, mostly native anglophone writers. It traces some promising avenues of research that challenge the cultural turn that gave birth to a new history of science thirty years ago. The present essay on this intimidating and stimulating monument of historiography does not spring from nowhere; it reflects the perspective of a French cultural historian of science of the early modern period. As an example of intelligent, innovative, reflexive, and always critical social and cultural history of science, the volume charts out fresh historiographical territories with great originality and imagination, inviting us to take its perspectives seriously. In this article, therefore, I would like to acknowledge the impressive collaborative work in this volume by giving a comprehensive overview of its academic research, while also pointing out some concerns.

**Writing History of sciences today: Poetics of Scientific Modernity**

By choosing to proceed through many short narratives, this multi-authored work refuses to endorse the structures of scientific revolution. As others have noted, broad overviews long tended to take the triumph or dominance and prestige of physics and mathematics in the twentieth century as their starting point, adopting these two disciplines as their paradigmatic core in order to create a great unifying narrative. In order to break with the intellectualist or idealist vision of the philosophical history of science that had been dominant among epistemological philosophers, historians who were more interested in experimental cultures and observational science tried to show how these approaches marked a dividing line between traditional (mainly Aristotelian) science and modern science. Their implicit hierarchy of disciplines and objects of science reflected the organisation of legitimate scientific fields in the twentieth century, creating a distinct category for mathematics, physics, astronomy, the life sciences and medicine. The invention of modern Science with a capital S among professionals, where Science had high cultural prestige, is said to have occurred in the nineteenth century and this book implicitly adopts this moment as its centre of gravity, although it includes significant passages on the early modern and mediaeval periods. To reinforce the grand narrative of scientific revolution, some historians of science fostered an approach to “scientific theories” that was buttressed by disciplinary genealogies (mainly mathematics, astronomy, physics, biology and medicine), while others tried to produce an archaeology of forms of scientific rationality. The latter retained the importance accorded to norms and method in breaking with more spontaneous practices of producing knowledge. Objectivity, quantification, observation, and experimentation appear as the pillars of the “scientific method” of modern science. This book does not challenge either the category of scientific disciplines as the core of its investigation or the imposition of boundaries separating them from those other disciplines that are regarded as less scientific. A comparison with other fields of knowledge requiring scientific rigour, such as law or geography, might have been productive – for example, historians have long noted that astronomy and natural history show similarities with ancient history and archaeology. Such blurring of categories facilitates an intellectual flexibility that is not bound to rigid definitions and which should be made explicit and theorised as a method of questioning science. Approaches to literary fiction and visual culture are similarly more complex than those dealing in “literature” or the “arts”, which appear as ahistorical categories (or solely contemporary from the nineteenth century). Meanwhile the relations between the natural and human sciences, such as anthropology, deserve more systematic attention (issues of terrain aside) in order to better situate problems of race.

Turning its back on an intellectual or disciplinary history of science, this *Companion* offers an overview based on an analysis of the practices and forms of scientific activity. The opening two chapters by Bernard Lightman and Lynn K. Nyhart introduce a collection of essays by some thirty scholars. While presenting itself as a continuation of both the eight volumes of the *Cambridge History of Science* edited by Lindberg and Numbers and the *Companion to the History of Science* published by Routledge in 1990 and edited by Olby, Cantor, Christie and Hodge, the present book recognises the considerable change in historiography since then. These introductions indicate that science is presented here as an activity that is at once work and cultural practice, ideal and material, and is not confined to the western world. The aim is thus to use these multiple points of entry and the overall structure of the book as a framework for a social and cultural history of science: "the science we depict is deeply embedded in its surrounding culture (even when scientists and spokesmen for science have argued otherwise)—yet that culture itself is typically not closed, but instead in constant exchange with the other cultures, feeding the wellsprings of scientific innovation, power, and conflict" (p. 17). The introductory chapter plays a crucial role in recasting cultural history of science in the aftermath of the American culturalist turn, placing into question certain conceits and naïve assumptions, rethinking discursive approaches, and adumbrating links between the cultural and the social that neither stifle cultural autonomy nor obliterate social reality.^[[2]](#footnote-2)^ Nyhart strategically insists on four different moves that structure the whole volume: “Constructing Scientific Knowledge, Socially”; “Doing Scientific Things with Scientific Things: Practice and Materiality”; “Moving Knowledge Around: Communication and Circulation”; “Scaling History of Science”.

The investigation of practices, places and the relations between science and societies goes some way to challenging the periodisation based on major figures inherited from the history of ideas. Writing history from below most of the time involves drawing on many short narratives and case studies, to the point where the field seems polarised between the grand narratives laid out in history textbooks and the tendency towards a cabinet of curiosities comprised of “exciting” cases. The historian of Chinese science Carla Nappi warns us of the consequences of case studies: “Historians have placed increasing emphasis on local case studies as a path toward a more polyvocal and encompassing narrative of science in global history. The logic of this seems to be that an agglomeration of these individual points should give us a more comprehensive history that respects local difference while weaving together individual stories into a common, global plot”.^[[3]](#footnote-3)^ In order to avoid the cabinet of curiosities, Bernard Lightman is also very clear about the nature and the scope of each chapter which should “be synthetic, midscale studies rather than microstudies” (p. 2). The debate seems confined to a question of narrative scale, while the omnipresence of scholarly storytelling goes unchallenged. The strategy is to stress the power of stories to offer counter-realities and a counter-narrative rather than to reject the charms of stories themselves. The first globalisation of science brought constraints that were quickly thrown off in the nineteenth century and more still in the twentieth, when science became global due to the internationalisation of discussions, practices, norms and standards, and through the establishment of a global governance of science by international institutions. In the last century science asserted its global nature still further through the development of fields such as ecology and climate science. The planet became an object of scientific investigation and argument. As scientific approaches to the Earth system separated into independent disciplines, global expertise became a matter of dispute. The more modest approach of this multi-authored *Companion* has avoided both revolutionary rhetoric and grand globalised gestures. The modernity of the history of science had a twin function. It was both a periodisation with the scientific revolution as its centre of gravity (thus a specific periodisation describing a cycle between the Renaissance and the Enlightenment) and a value (modernity drew on a normative, positive definition of science as progress). This representation is now in crisis. The latter function has been strongly attacked by post-modern and post-colonial theory, while the former is losing its function for historical orientation through the trend towards fragmentation and excessive dilation. What kind of revolution lasts several centuries? The short stories on offer here are the antidote to any return of the grand traditionalist narrative of the now-globalised scientific revolution. While this book’s centre of gravity remains the invention of modern science in the nineteenth century, this is not unanimously accepted by scholars and some of the contributors, such as Peter Dear, have written elsewhere on the specificity of early modern science.^[[4]](#footnote-4)^ There have been many attempts to nuance the periodisation, and historiographic frameworks have been reshaped by explorations of *classical science* (Halleux and Blay) and *baroque science* (Ofer Gal and Raz Chen-Morris) or drawing on the Iberian historiographic paradigm.^[[5]](#footnote-5)^ The absence here of any discussion of these different paradigms and historiographic approaches is symptomatic of a desire to retain the unity of the history of science in terms of its questions and method linked to the anglophone academic world.

**The primacy of roles: a sociological perspective**

From the outset the reader is struck by the attention paid to the actors of science, both human and non-human (instruments, collections etc.). How is this sociology of actors to be conducted? In Part I of the book the contributors use the category of social roles to study scientific practices from Antiquity to the present day. This is a significant choice, positive in its exclusion of other approaches and negative in confining the description to a network of concepts that is not always well suited to the analysis of practices.

The notion of roles has a long history in sociology and relates to the idea that society is made up of interactions between positions that have been or are yet to be constructed. It can be linked to a sociology of the rules defining a repertoire of cultural models or to an attitude, a social representation, or even a posture. The definition of the role is thus dynamic (in the theatrical sense of the word), since a role must be taken on; it is not imposed or inherited. The term has served as a pillar of Mertonian sociology. It can lead to conflict through the desire to stabilise roles and turn them into status. In recent decades the concept of persona has found favour with many historians of science seeking to establish the archetypal representations available to a savant in any given period, and historians have often been torn between the categories of vocation and profession in describing an exceptional scientist. The sociology of social roles avoids the vague category of the persona and its timeless dimension. For the architects of this book, scientific work gave rise to a division of labour, hierarchisation and specialisation. This represents a move away from the two more traditional approaches, one celebrating genius through biographies (Galileo, Newton, Einstein), the other a more anonymous view of the scientific worker so dear to the sociology of science. Part I of this book adopts an approach of scientific singularity by considering the different social roles that have made up the persona of the savant from Antiquity to the contemporary period. The list comprises the predictable figures of the savant – male or female – alchemist, natural philosopher or naturalist, instrument maker and artisan, who are joined by less predictable actors such as illustrators, go-betweens, travellers and translators, as well as the subjects and victims of scientific and medical experiments (prisoners, slaves, patients, children) and amateurs. The tensions between “professional” and “amateur”, amplified by those between men and women or teachers and artisans, reflect well-established dichotomies between public and private (p. 75) and reader and experimenter (chapter 5). The theory of roles is clearly linked to an effort of disciplinary and sociological demarcation and a process of legitimation. This is particularly true at the social frontiers of the world of science. In the words of Iwan Rhys Morus, “the problem of scientific authority has always been closely bound up with the identity of the knowledge-maker” (p. 97). The point of the exercise has thus been to enrich the description of savant worlds, expanding the list of actors and giving visibility to the “invisible technicians” in accordance with social history from below, gender studies and subaltern studies. Rhys Morus concludes: “It is not a historian’s task to judge how demarcations between scientific and non-scientific work should be made. It is a historian's task to enquire into how and why such discriminations are historically constructed” (p. 108). But this social invisibility is a construction at once historiographic and historical, which has sometimes seen the foregrounding of women, artisans and more recently go-betweens in the processes of knowledge production. With go-betweens and the subjects of experiments, Kapil Raj and Anita Guerrini have taken the original approach of placing themselves at the extreme edge of the sociological approach to scientific agency by analysing “science” without, despite, and beyond “scientists”. Through these new studies, it is the concept of actor itself that is radically questioned in its classical definition as intentional, strategic and calculating. Discussing the historiography of the amateur, Katherine Pandora clearly shows the importance of shedding a normative view of science in order to grasp the dynamics of appropriation. These approaches are no longer regarded by default as histories of the process of disciplinarisation and professionalisation of science, but as places of innovation and porous zones of a cultural history of science (p. 146-147).

One of the contributions of this first part is that it highlights technologies and strategies of social visibility and invisibility and also establishes a collective view of scientific work. But do social roles offer the best framework for describing and interpreting practices? We might wonder whether this sociology of science would have gained from the integration of other sociological models, notably pragmatic sociology, in considering issues of practice and longevity, observational scale and recognition.^[[6]](#footnote-6)^ Social history can go beyond social constructed roles and their attendant reputations and social and cultural representations, and also beyond reputations of “greatness”.^[[7]](#footnote-7)^ The sociology of science has not entirely managed to eliminate the vocabulary of “genius”, but it has tried to take it seriously (in regard to the aura, the relationship to collective intelligence, etc.).

A second criticism stems from contemporary analyses in which this network of stable social roles is attacked. Scientific capitalism transformed radically the social world of science by reversing the polarity between Research and development activities in Private Companies and Universities. While the chapters on “The man of science” and “The professional scientist” remain prisoners to questions of the individual and the community, the book overlooks recent research on contemporary scientific work. Since the 1950s the pursuit of science has been no longer regarded as a creative intellectual activity but as disciplined, organised work and as a “scientific human resource” to be quantified and planned. Historians such as Steven Shapin have recently offered a different picture, focusing on the scientific technician.^[[8]](#footnote-8)^ Many statistical reports were commissioned by governments in the United States and Europe to assess recruitment policies. They modified the definition of the scientist, which moved in American terminology from the qualified scientist and engineer to the “entrepreneur scientist” associated with the emergence of electronics start-ups in the 1970s. In other circles scientists left the isolation of their laboratories to provide governments with expert opinions in the new context of environmental and health risk management or in the establishment of infrastructure. Starting from a post-Mertonian vision of scientific work, Part I subverts the grand narrative of professionalisation but without escaping it or saying what professionalisation means, when this concept and that of discipline have been extensively revisited in sociology by Andrew Abbott. It is in the book’s other parts that we will find useful ways into understanding the new practices of scientific capitalism (see Paul Lucier’s chapter in the volume).

**A triple material turn**

Strongly marked by the cultural history of science of the 1990s, this book reflects the material turn taken by the history of science in the last decade. It closes with a Part IV entirely devoted to material scientific cultures, offering a different narrative around a focus on instrumentation in all its forms. Instruments are described in detail within their different contexts: from clocks to weights and measures (Hector Vera), calculating machines (Matthew L. Jones), microscopes (Boris Jardine), telescopes (Jim Bennett) and more classically specimens and collections, spectroscopes and diagrams including maps, tables, graphs (Charlotte Biggs) and models (Joshua Nall and Liba Taub). Such tools of science are regarded as “total social facts” (469). Instruments provide the framework for work and experimental culture. Since the 1980s a whole school in the sociology of science has sought to develop our understanding of the material culture of scientific work. The first aim was to bring symmetry to the analysis by acknowledging the full importance of objects such as instruments, apparatus and machines in the processes of constructing scientific facts. As noted by Ian Hacking, “we create an apparatus that produces data that confirm the theories; we judge the apparatus according to its capacity to produce data that sticks”.^[[9]](#footnote-9)^ The contributions here do not all take the same approach and are organised around two centres of interest arising out of the material history of instruments. One is metrology and the social history of quantification. A group of studies of the 1990s considered the moral dimension of measuring, among them those of Graeme Gooday in Leeds and Simon Schaffer in Cambridge on the emergence of standards and metrology. The other focus emphasises the visual and material cultures of science as important for an anthropology of science and intellectual technologies, seeking to describe “mindful hands”.^[[10]](#footnote-10)^ While the former approach strongly rooted in the social history of disciplinary institutions and labour brought the history of science closer to E.P. Thomson and Michel Foucault, the latter placed the emphasis on material technologies in a dialogue with the anthropology of techniques and the history of art (André Leroy-Gouran; Alfred Gell; Sveltana Alpers).

This material history of science emphasises the performative constitution of a certain reality of natural phenomena in the scientific world. As most of the chapters in this part remind us, the notion of audience is key here. Part IV—but also chapter 19 on “Commercial sciences” by Paul Lucier—constitute the most original contributions of this book, offering detailed discussions of the available reading and critical thinking about materiality, materials, tools and objects. By connecting science to the market, the volume acknowledges the importance of economy and commodification in the making of scientific practices, not because sciences should be driven by the market of applied innovation, but because market culture embraced sciences as a production of goods. Here we find a strong link between history of science and history of material culture as consumption which displace the common definitions of the material world and complicate the relations between sciences and things. However, Craciun and Schaffer have recently encouraged historians of sciences to complicate their analytical categories: “Things may be the wrong term here. We might as well speak of objects, artifacts, waste, commodities, specimens, ephemera, relics, artworks, instruments, souvenirs. Each of these terms is bound up with specific economic models, of making, seizure, collection, appropriation, storage and exchange”.^[[11]](#footnote-11)^

**Places and spaces of science: a spatial history**

It is no coincidence that Part II of the book is devoted to places and spaces. As we know, the history of science has long been a history of institutions, no longer confined to describing institutional complexity or listing the equipment of scientific modernity (laboratory, observatory, cabinet of curiosity, botanical garden), but now also interrogating the dynamics of the territorialisation of science. The pioneering theories of Steven Shapin and Adi Ophir, and the work of the historical geographers Charles Withers, David Livingstone, Miles Ogborn, and Diarmid A. Finnegan in this volume, revealed places as sites of both the production of science and urban culture. In France, the monumental *Lieux de savoirs* (2007-2011) edited by Christian Jacob proposed rethinking the notion of intellectual work by interrogating places of knowledge from the perspective of a comparative anthropology of literate worlds. This enterprise led to the construction of a field of research that is at once transperiodic and transdisciplinary, breaking the boundaries of scales of comparison from one continent to another. The *Companion*’s undoubtedly more modest enterprise is to make an inventory of emblematic places, starting with the mediaeval university (Antiquity is strikingly absent from Part II). All the same, we cannot avoid noting that the list of places remains fairly traditional, focusing on spaces of research (universities, laboratories, museums, botanical gardens, courts) to the detriment of analyses at other scales (for example the city) or discussions of regional spatial dynamics (the Atlantic, the Indian Ocean, the Pacific, empires). Each author makes an effort to show how each place organises scientific activity in its own way. The production and circulation of knowledge involve particular modes of spatial inscription and territorial logic. But each place of knowledge also defines the limits of its influence or jurisdiction, to use a legal metaphor, which can lead to boundary disputes between academies and universities, or today between public bodies and business. For instance, the court as studied by Bruce Moran offers a particular context operating through courtly performance, visual knowledge and curiosities, in which he interrogates both the utilitarian dimension of science as manifested in manufacturing and empire and the gender frontier through the recruitment of women. It would, however, be possible to go beyond the issue of patronage. The court as a scientific laboratory could also be linked to environmental questions and to the expression of ecological absolutism.^[[12]](#footnote-12)^ Architectural issues are considered. The monumentalisation of places of knowledge in the nineteenth century tells us much about the prestige of science and its competition with less temporal powers. In exploring all the meanings of place, from location to belonging, this part discusses many methodological and theoretical approaches.

**Communicating Science: a post-Habermasian moment?**

Another powerful dynamic in the book involves revisiting the “diffusion” of modern science through the concepts of circulation and translation. Starting from the division between public and domestic space (Donald L. Opitz), the book deals directly with the question of separate spheres and complexifies the Habermassian perspective by approaching it through concepts such as “commercial science” and “popularisation” as used in research on the nineteenth century. More broadly, the book returns to key concepts of the historiography of the last twenty years, such as the “Field” (Kohler and Vetter), which goes far beyond its contrast with the laboratory to become a paradigm that is used to build hierarchies and redefine practitioners. This chapter reveals the boundless expansion of field sciences and the mobility of the concept.

Following on from Part II, Part III focuses on communication and so also on forms of circulation, providing a useful overview of work on the history of manuscripts, printed books and correspondence, from the Republic of Letters to the digital humanities (Brian Olgivie). Beyond offering sources and archives, these chapters are crucial for understanding how a focus on places has given way to an emphasis on circulation. From translations to savant periodicals, lectures to textbooks, and including film, radio and television, these authors take communication seriously. The last thirty years has seen the development of histories of books and reading and also of scientific institutions, sociabilities, intellectual techniques, education and educational practices, partially eclipsing an earlier focus on writing with the “sacralisation” of visual and oral communication. These different means of communication should perhaps be contrasted in order to reveal the processes of relegating or promoting one over another. I will suggest two possible ways the conversation has developed.

First, the triumph of one means of communication over another is never methodologically established, as Françoise Waquet recalled in relation to savant orality: “these spoken word activities that constitute a notable element […] of the use of scientific time have been historiographically confined to the mute context of intellectual life, that of the institutions of knowledge, when they have not been reduced to the anecdotal level of picturesque detail decorating a biographical narrative”.^[[13]](#footnote-13)^ This “disappearance” of savant orality as a historical problem can be blamed on the sacralisation of the printed or manuscript book and indeed of traces and signatures.

Second, practices of communication are always described in relation with their urban context according to an Habermasian paradigm. The central dimension of the metropolis (which should be added in the list of places) was also crucial for the debate about the role played by modern sciences in the making of an Habermasian public sphere. An avenue of research very common during the 1990s was to explore the public sphere of science, to map the urban cultures of sciences already studied, thus also underlining the distribution of networks of exchange woven through these cities. This practical orientation, certainly, led historians to retain the ordinary criteria of an intellectual centre: editorial center, university, points of exchange in the Republic of Letters; urban sociabilities (salon, circles, clubs), but also urban spectacles of science. The chapters on public lectures, journals and periodicals, textbook, film, radio and television show the capacity of an urban milieu to exploit and diversify its forms of knowledge, to reshape scientific authority and to displace the demarcation line. By visualizing sciences, using body language (Finnegan, p. 419), scientists have used the metropolitan public sphere to diffuse sciences. If metropolitan scientific life is described as the intensification of sociability, local interactions and facilities, we must therefore reinterpret *hauts lieux* such as court visits or the Academy of Sciences as examples of scientific performances, and so consider to what extent metropoles correspond with the concept of hyper-places (*hyper-lieux)*.^[[14]](#footnote-14)^ The production of local knowledge or situated knowledge in the metropolitan context is largely dependent on what Arjun Appardurai called a “global production of locality”.^[[15]](#footnote-15)^ Studies looking at credibility and the authority of nature in the city have stressed the importance of public negotiation in the maintenance of long-term networks.^[[16]](#footnote-16)^ However, the debate about sciences and democracy shows how difficult it is to deal with communication as a way of making sciences public. The new framework of democracy studies not only considers diffusion, spokesmen, and freedom of speech, but also presents the problem of transparency and the right for publics to know. According to John Urry, modern democratic societies are experiencing the development of a culture of offshoring as a mechanism to elude transparency and to produce strategic opacity.^[[17]](#footnote-17)^ What about sciences in this new context? Is it possible to revisit the Habermasian public sphere of sciences with new analytical tools?

**Report from the edges of the World**

Even though central to the book, the spatial turn could have been more broadly represented. More attention could have been paid to spatial dynamics, particularly in non-European cultural zones, without slipping into conventional divisions (science in India, China, science and the Ottoman empire, etc.). Historians of science on other continents were left to position their objects of study (Arab, Chinese, Indian science, etc.) in relation to the standard. However, to better historicise science in the modern period, historians of science have since abandoned such simplifications and chosen to highlight the epistemological tension between science and knowledge. As Lynn K. Nyhart subtly puts it,

[A]n older predominant history of science might be captured by the image of a tree of scientific ideas rooted in the case of Western Culture (perhaps extending downward earlier to ancient Egypt and Babylonia); the task of the historian of science was to trace the tree’s growth and branching. Today a more fitting image would be of the history of science as a densely tangled bank of people and material things teeming with social, cultural, economic, and religious life, that covers the globe. The historian's task now is to tease out how certain forms of knowledge and practice within this mass of activity came to be understood as “science;” what has sustained science socially, culturally and materially; and who has benefitted and who has suffered in its formation (p. 7).

There are two reasons for this approach: firstly, it permits a modification of the chronology of the invention of modern science, confining it to the nineteenth century. Secondly, a focus on science and knowledge “provincialises” Europe as the place that invented a western form of organisation of knowledge about nature or a specific rationality known as science, allowing questions of the globalisation of knowledge to remain open. Something different happened in Eurasia, China and the Moghul empire that is neither simply a matter of scientific transfer nor of colonial development. They had a trajectory of their own. Some years ago, Jurgen Renn observed: “More importantly, non-Western epistemic practices are also considered without being immediately gauged against the standards of established Western science”.^[[18]](#footnote-18)^ Renn also critiqued the proliferation of descriptions of processes and contexts that has led the image of science to fragment. In his view, “the history of science can only be understood against the background of a global history of knowledge”. Probably, we can regret that the *Wiley Blackell* *Companion* has little to say on these discussions of western singularity or on the end of exceptionalism, although these issues seem very present in today’s journals in the history of science. The globalisation of science or the transnationality of science are only different aspects of the long globalisation of knowledge and they are linked to particular processes of political, economic, and cultural globalisation or internationalisation.

Rather than considering the globe as a natural entity, some historians have recently attempted to denaturalize it, to show that the superposition between the globe and the world is a recent view and that globalization was the result of a long and contradictory process which involved merchants, cartographers, missionaries and mirrored different projects^[[19]](#footnote-19)^: “If Postcolonial studies has succeeded in provincialising Europe, it still exerts one imperial dominion in its full and undisputed splendor, namely this strange idea that the Globe is the equivalent of the natural world. My argument is that there has been confusion between the Globe and the Earth”.^[[20]](#footnote-20)^ The contribution of this book, with its firm focus on the west, is less ambitious. Two chapters that escape this critique and deserve the reader’s attention are those by Raj on “Go-betweens”, and by Carla Nappi and Marwa Elsraki on translation. These essays, located respectively in Parts I and III, offer a coherent methodological vision. Against a diffusionist and evolutionist paradigm that places modern western science at the pinnacle of scientific achievement, they start from practices and actors that mediate different scientific cultures, but without reifying them. Drawing on hybrid and connected history and diaspora studies, the aim is to better understand instances of scientific interaction, circulation and travel and processes of translation and integration: “These contributions thus stress the historical contingency of these practices and mutations in knowledge and taxonomies introduced by movement itself” (44). Linguistic translation and issues of scientific language are far from settled. Yet, like current debates in comparative literature around the definition of world literature, historians of science have recently launched into these discussions.^[[21]](#footnote-21)^ This is a way of working not only on spaces but also on the time and historicity of scientific concepts and practices.

**Beyond Culturalism: where is the anthropological and environmentalist turn?**

Following research into an anthropology of nature (Philippe Descola, Tim Ingold, Bruno Latour) and under pressure from environmental history, the last decade has seen the emergence of a new history of science focusing on subjects such as natural disasters, the Anthropocene period and scientific ecology as a discipline. The history of science seems to be giving way to a history of nature. The obsession of the historians of science of yesterday with contrasts between tradition and innovation or between religion and secularisation has now been replaced by a focus on the constitution of western naturalism between the Renaissance and the French Revolution (Philippe Descola) or efforts to identify the contribution of scientists to establishing the Anthropocene.^[[22]](#footnote-22)^ A new historiographic cooperation between the history of science, environmental history, and anthropology is reflected in the convergence of investigations around nature and the environment. This anthropological turn is no longer confined to the ethnography of laboratories, as it was in the 1970s, but is rooted in a concern to better understand the relationship of science to the environment, animals and objects, and to close and distant forms of rationality. If the cultural history of science was eager to broaden the list of scientific actors or practitioners within society (women, artisans, amateurs, and now slaves, go-betweens, non-humans), new researches draw the attention of historians of sciences to discussions among geographers, sociologists and anthropologists on environmental conditions. Such sources may be useful for historians of sciences in order to avoid any culturalist and reified concepts of cultural identity.^[[23]](#footnote-23)^ Is it possible to have a non-discursive or non-representational history of science that doesn't fall into the culturalist trap? ^[[24]](#footnote-24)^ How can we interpret the absence of these new centres of interest from this book? Does it reflect a resistance to new historiographical approaches or simply a disengagement from the environmentalist tropism of the history of science?

This gap seems to me characteristic of a long-term editorial project which, despite its intrinsic strengths, has gradually fallen out of step with current developments in historiography. Or perhaps we should see it as a form of tacit collective defence of the cultural history of science in which this book is grounded, by expanding the borders of cultural history far beyond the cultural turn. The rematerialization of science pursued in this volume is not only matter of instrumentation, location and social connection; it is not only related to material culture. Indeed, the ontological turn obliges us to rethink our conceptual tools.^[[25]](#footnote-25)^ It helps to better understand the tension between a natural history of nature and a human (or cultural) history of environment. But obviously the volume should not simply endorse the school of thought but provide critical engagement. As Steve Woolgar and Javier Lezaun suggested: “‘Ontology’ has sometimes been used as a sort of signifier to claim a more thorough-going or insistent form of deconstruction, but it remains unclear how claims about the ontological composition of the world differ from more conventional propositions about the social construction, co-production, or performative constitution of a certain reality”.^[[26]](#footnote-26)^ For many historians of sciences today, it is a way of imposing a new analytical approach that includes a multi-naturalism.^[[27]](#footnote-27)^

**The Curse of Technosciences: The end of Science?**

We started our review with the questioning of grand narratives and the fall of the scientific revolution, but we need also to question the end of the story. What about our current definitions of scientific practices?^[[28]](#footnote-28)^ This is not solely a matter for historians of sciences as custodians of the idea of science. This idea is no longer a matter of consensus. This book has less to say about the late twentieth century, yet the emergence and globalisation of technoscience asks profound questions of the history of science. Today the notion of public science itself is no longer self-evident and the need to democratise scientific life seems more pressing than ever. Since the 1970s, the history of science has analysed the reception of science by society, either by investigating public participation and the audience for the experimental validation of scientific propositions, or by focusing on the new media (mainly the scientific press) that appeared at the turn of the eighteenth and nineteenth centuries. A movement of criticism of sciences that first emerged before the Second World War criticised working conditions in laboratories and more generally opposed elitism in science, following internal criticisms of the risks that scientific research was becoming “militarised” (the Manhattan project). Meanwhile the movement for the public understanding of science in the English-speaking world sought to break down barriers and integrate science into the democratic life of modern societies. The public sector became involved in this process through the proliferation of science museums and bodies to support scientific culture. The French physicist and epistemologist Jean-Marc Lévy-Leblond has provocatively suggested that modern science has perhaps passed away, given the extent to which technology and the market in innovation now dictate and shape research agendas. **^[[29]](#footnote-29)^** He notes that in the course of its long history science has sometimes disappeared – he cites the Roman Empire as an example – in favour of technology.^[[30]](#footnote-30)^ Whether in relation to debates over the Anthropocene period and ecological crisis, “neo-liberal” science, or the democratisation of sites of production, the historians in this volume make history an exercise of lucidity even within the wider context of often presentist visions of science which count the past for little. No doubt the book could have made more of the plurality of current historiographies on the world of science, including those heterogeneous historiographies that constitute minority voices within the discipline. However, it remains a work of conviction and indisputable success in promoting the history of science, not as an ultra-specialised field but as a crucial mode for the interpretation of societies in their relations to both knowledge and nature.

**References**

Alder K. (2013). The History of Science as Oxymoron from Scientific Exceptionalism to Episcience. *Isis*, 104, p. 88-101.

Appadurai A. (1997). *Modernity at Large: Cultural Dimensions of Globalizations*. Delhi: Oxford University Press.

Barthe Y. and al. (2013). Sociologie pragmatique: mode d'emploie. *Politix*, 103, p. 175-204.

Bensaude-Vincent B. (2009), *Les vertiges de la technoscience. Façonner le monde atome par atome*, Paris: La Découverte.

Bonnell V. and Hunt L. (eds.) (1999). *Beyond the Cultural Turn. New Directions in the Study of Society and Culture*. Berkeley: University of California Press.

Chemla K. and Fox Keller E. (eds.) (2017), *Cultures without Culturalism. The Making of Scientific Knowledge*, Durham and London: Duke University Press.

Craciun A., Schaffer S. (eds.) (2016). *The Material Cultures of Enlightenment Arts and Sciences*. London: Palgrave.

Daston L. and Park K. (2006) (eds.). *The Cambridge History of Science*, vol. 3: Early Modern Science, Cambridge, CUP.

Dear P. (2012), "Historiography of a not-so-recent science", *History of Science*, 1: 197-211.

Delbourgo J. (2017). *Collecting the world. The life and curiosity of Hans Sloane*. London: Penguin, Allen Lane.

Descola P., (2013) (2005) *Beyond Nature and Culture*. Chicago: Chicago University Press.

Elshraky M. (2010). When Science Became Western. Histiographical reflections. *Isis*, 101, 98-109.

Fransen S., Hodson N., and Enenkel K., eds. (2017). *Translating Early Modern Science*. Leiden: Brill.

Gordin M. (2015). *Scientific Babel: The Language of Science from the Fall of Latin to the Rise of English*. Profile Books.

Hacking I. (2003). *Representing and Intervening: Introductory Topics in the Philosophy of Natural Science*. Cambridge: Cambridge University Press.

Hicks D.J. and Stapleford T.A. (2016), “The Virtues of Scientific Practice: MacIntyre, Virtue Ethics, and the Historiography of Science,” *Isis* 107, no. 3, p. 449–72.

Harman P. (2009). *The Culture of Nature in Britain, 1680-1860*. New Haven: Yale University Press, 2009.

Jacob C. (2008-2011). Lieux de savoir. Paris: Albin Michel, 2 vols.

Latour B. (2016). *Onus Orbis Terrarum*: About a Possible Shift in the Definition of Sovereignty. *Millennium. Journal of International Studies*, vol. 44 (3), p. 305-320.

Lehoux D. (2012). *What Did the Romans Know? An Inquiry into Science and Worldmaking*. Chicago: Chicago University Press.

Lévy-Leblond J.-M. (2012). *Le grand écart. La science entre technique et culture*. Paris: Éditions Manucius.

Lilti A. (2017). *The Invention of Celebrity*. Trans. Lynn Jeffress. Cambridge: Polity.

Lussault M. (2017). *Hyper-lieux: les nouvelles géographies politiques de la mondialisation*. Paris: Le Seuil.

Moatti C. (1997). *La Raison de Rome. La naissance de l'esprit critique à la fin de la République*. Paris: Le Seuil Publisher.

Nappi C. (2013). The Global and Beyond. Adventures in the Local Historiographies of Science. *Isis*, 2013, 104, 102-110.

Ofer G. and Chen-Morris R. *Baroque Science*. Chicago: Chicago University Press, 2012.

Pestre D. (2015) (ed.). *Histoire des sciences et des savoirs*. Paris: Le Seuil, 2015, 3 vols.

Pestre D. (ed.) (2014), *Le gouvernement des technosciences. Gouverner le progrès et ses dégâts depuis 1945*. Paris: La Découverte.

Pimentel J. and Pardo-Tomas J., And yet, we were modern. The Pardoxes of Iberian science after the Grand narratives. *History of Science*, 2017, 55 (2), 133-147.

Porter R. (2003) (ed.), *The Cambridge History of Science*, vol. 4: Eighteenth Century Science. Cambridge, Cambridge University Press.

Quenet G. (2014). *Versailles : une histoire environmentale*. Paris: La Découverte.

Renn J. (2015). The History of Science and the Globalization of Knowledge. in T. Arabatzis et al. (eds.), *Relocating the History of Science*. Boston: Boston Studies in the Philosophy and History of Science, 2015 (pp. 241-252).

Roberts L., Schaffer S. et Dear P. (eds.) (2007). *The Mindful Hand: Inquiry and Invention from the Late Renaissance to Industrialization*, Amsterdam.

Schaffer S. (2003), “What is Science?”, in. Krige and Dominique Pestre (eds.), *Companion to Science in the Twentieth Century*. London: Routledge, (p.27-41).

Shapin S. (2008). *The Scientific Life. A Moral history of a Late Modern Vocation*. Chicago: Chicago University Press.

Urry J. (2014). *Offshoring.* Cambridge*,* Polity.

Van Heurn B., Leydesdorff L., Wyatt S. (2013). Turning to ontology in STS? Turning to STS through 'ontology'? *Social Studies of Science*, 43 (3), 341-362.

Waquet F. (2003). *Parler comme un livre. L'oralité et le savoir, XVIe-XXIe siècles*. Paris: Albin Michel.

Woolgar S. and Lezaun J. (2013). The wrong bin bag: A turn to ontology in science and technology studies? *Social Studies of Science*, 43 (3), 321-340.

1. Daston and Park (2006); Roy Porter (1999); Pestre (2015). [↑](#footnote-ref-1)
2. Bonnell and Hunt (1999). [↑](#footnote-ref-2)
3. Nappi (2015). [↑](#footnote-ref-3)
4. Dear (2012). [↑](#footnote-ref-4)
5. Pimentel and Pardo-Tomas (2017) [↑](#footnote-ref-5)
6. Barthe (2013). [↑](#footnote-ref-6)
7. Lilti (2017). [↑](#footnote-ref-7)
8. Shapin (2008). [↑](#footnote-ref-8)
9. Hacking (2003). [↑](#footnote-ref-9)
10. Roberts, Schaffer and Dear (2007). [↑](#footnote-ref-10)
11. Craciun, Schaffer (2016), p. 3. [↑](#footnote-ref-11)
12. Quenet (2014). [↑](#footnote-ref-12)
13. Waquet (2003), p. 8. [↑](#footnote-ref-13)
14. See on ‘hyper-places’, Lussault (2017). [↑](#footnote-ref-14)
15. Appadurai (1997). [↑](#footnote-ref-15)
16. Recently a good example is Delbourgo (2017). [↑](#footnote-ref-16)
17. John Urry, *Offshoring,* Cambridge*,* Polity, 2014. [↑](#footnote-ref-17)
18. Renn (2015), p. 242. [↑](#footnote-ref-18)
19. Romano (2016), Latour (2016). [↑](#footnote-ref-19)
20. Latour (2016), p. 307. [↑](#footnote-ref-20)
21. Gordin (2016); Fransen, Hodson and Enenkel (2017). [↑](#footnote-ref-21)
22. Descola (2005). [↑](#footnote-ref-22)
23. A good example of a culturalist cultural history of nature could be found in (Harman, 2009). [↑](#footnote-ref-23)
24. Chemla K. and Fox Keller E. (eds.) (2017). [↑](#footnote-ref-24)
25. For a critical assessment, see Woolgar and Lezaun (2013) and Van Heurn, Leydesdorff, Wyatt (2013). [↑](#footnote-ref-25)
26. Woolgar and Lezaun (2013), p. 322. [↑](#footnote-ref-26)
27. Latour (2016). [↑](#footnote-ref-27)
28. Hicks and Stapleton (2016). [↑](#footnote-ref-28)
29. Lévy-Leblond (2012). [↑](#footnote-ref-29)
30. Lehoux (2012), Moatti (1997). [↑](#footnote-ref-30)
